# Vitamin D receptor upregulates lncRNA *TOPORS-AS1* which inhibits the Wnt/β-catenin pathway and associates with favorable prognosis of ovarian cancer

**DOI:** 10.1038/s41598-021-86923-7

**Published:** 2021-04-05

**Authors:** Yuanyuan Fu, Dionyssios Katsaros, Nicoletta Biglia, Zhanwei Wang, Ian Pagano, Marcus Tius, Maarit Tiirikainen, Charles Rosser, Haining Yang, Herbert Yu

**Affiliations:** 1 University of Hawaii Cancer Center, 701 Ilalo Street, Honolulu, HI 96813 USA; 2 Department of Surgical Sciences, Gynecology, AOU Città Della Salute, University of Torino, Turin, Italy; 3 Division of Obstetrics and Gynecology, Department of Surgical Sciences, University of Torino School of Medicine, Mauriziano Hospital, Turin, Italy; 4 Department of Molecular Biosciences and Bioengineering, University of Hawaii at Manoa, Honolulu, HI USA; 5 Department of Surgery, Cedars Sinai Medical Center, Los Angeles, CA USA

**Keywords:** Cancer, Biomarkers, Molecular medicine, Oncology

## Abstract

Long non-coding RNAs (lncRNAs) have important biological functions, but their involvement in ovarian cancer remains elusive. We analyzed high-throughput data to identify lncRNAs associated with ovarian cancer outcomes. Our search led to the discovery of lncRNA *TOPORS Antisense RNA 1* (*TOPORS-AS1*). Patients with high *TOPORS-AS1* expression had favorable overall survival compared to low expression. This association was replicated in our study and confirmed by meta-analysis. In vitro experiments demonstrated that overexpressing *TOPORS-AS1* in ovarian cancer cells suppressed cell proliferation and inhibited aggressive cell behaviors, including migration, invasion, and colony formation. Analysis of tumor cell transcriptomes indicated *TOPORS-AS1*′s influence on the Wnt/β-catenin signaling. Additional experiments revealed that *TOPORS-AS1* increased the phosphorylation of β-catenin and suppressed the expression of *CTNNB1*, disrupting the Wnt/β-catenin pathway. Our experiments further discovered that vitamin D receptor (VDR) upregulated *TOPORS-AS1* expression and that inhibition of β-catenin by *TOPORS-AS1* required a RNA binding protein, hnRNPA2B1 (heterogeneous nuclear ribonucleoprotein A2B1). Taken together, these findings suggest that *TOPORS-AS1* may behave like a tumor suppressor in ovarian cancer through interrupting the Wnt/β-catenin signaling and that VDR upregulates the expression of *TOPORS-AS1*. Assessing *TOPORS-AS1* expression in ovarian cancer may help predict disease prognosis and develop treatment strategy

## Introduction

Ovarian cancer is a lethal gynecological disease, and the 5-year survival rate is dismal, only 47%^1^. As most patients are diagnosed with an advanced disease, few effective therapies are available for ovarian cancer treatment. Currently, there are no reliable tests available to detect ovarian cancer early. Biomarkers for accurate prediction of disease outcome and treatment response are also limited. Studies of ovarian cancer mechanisms have largely focused on proteins and their coding genes. Since protein-coding genes account for only 2% of the human genome^2^, our understanding of ovarian cancer development and progression from the genome perspective remains inadequate. Lately, long non-coding RNAs (lncRNAs), which contain 200 nucleotides or more with no or limited protein-coding potential, have been recognized to have crucial roles to play in regulation of biological functions and cellular activities. Emerging evidence also suggests that lncRNAs play a role in cancer, including ovarian cancer^3–9^. Understanding the involvement of lncRNAs in ovarian cancer may provide new insights and offer novel opportunities for diagnosis, prognosis, and treatment. In search for lncRNAs which might be implicated in ovarian cancer, we analyzed ovarian cancer survival in association with many lncRNAs whose expression data are available online in public databases. Our search led us to find the expression of *TOPORS Antisense RNA 1* (*TOPORS-AS1*) significantly associated with overall and progression-free survival of ovarian cancer.

Vitamin D receptor (VDR) is a nuclear receptor which binds to its ligand, 1, 25-dihydroxyvitamin D_3_, and acts as a transcription factor in regulating important cellular activities and functions, such as immunity, cell proliferation, differentiation and apoptosis^10^. VDR is known to have strong anti-tumor properties, including suppression of proliferation and inflammation, promotion of differentiation and apoptosis, as well as inhibition of angiogenesis and tumor cell invasion and metastasis^11^. VDR is expressed in the ovaries and is involved in the regulation of estrogen biosynthesis and aromatase activity^12–14^. Increasing VDR expression in ovarian cancer cells inhibits cell proliferation and tumor growth^15^. Evidence also suggests that VDR may interact with lncRNAs to exert its functions^16^. VDR was found to increase the expression of *MEG3* (maternally expressed gene 3), a tumor suppressor in ovarian^17^ and colorectal cancers^18^. In this report, we discussed our discovery of lncRNA *TOPORS-AS1* whose expression was regulated by VDR, associated with ovarian cancer survival, and able to inhibit the Wnt/β-catenin signaling.

## Results

### Association of *TOPORS-AS1* expression with disease stage and patient survival

Data from our study (Table 1) showed that *TOPORS-AS1* expression was higher in early (Stage I or II) than late stages (Stage III or IV) of ovarian cancer (p = 0.0046). *TOPORS-AS1* expression was not different between serous and non-serous tumors, nor changed by tumor grade or patient age at surgery. High expression of *TOPORS-AS1* was found to be associated with favorable overall survival, and the association remained significant after adjusting for patient age, disease stage, tumor grade and histology (Table 2). In comparison to those with low *TOPORS-AS1*, patients with high expression had over 40% reduction in risk of death (HR = 0.58, 95% CI 0.34–0.99, p = 0.046). Risks for disease progression, however, were not significantly different between patients with high and low *TOPORS-AS1* expression regardless of their clinicopathological features.

**Table 1:** Associations of *TOPORS-AS1* with clinicopathological factors of ovarian cancer.

Variables	Total no. (N = 242)	*TOPORS-AS1* expression			P value
		Low, no. (%)	Mid, no. (%)	High, no. (%)	
**Age at surgery**					
≤ 59.08	114 (47.70)	36 (31.58)	40 (35.09)	38 (33.33)	0.92
> 59.08	125 (52.30)	42 (33.60)	41 (32.80)	42 (33.60)	
**Disease stage**					
I–II	65 (27.08)	16 (24.62)	17 (26.15)	32 (49.23)	**0.0046**
III–IV	175 (72.92)	64 (36.57)	64 (36.57)	47 (26.86)	
**Tumor grade**					
1–2	64 (26.56)	20 (31.25)	22 (34.38)	22 (34.38)	0.92
≥ 3	177 (73.44)	60 (33.90)	60 (33.90)	57 (32.20)	
**Histologic type**					
Non-serous	145 (59.92)	46 (31.72)	52 (35.86)	47 (32.41)	0.72
Serous	97 (40.08)	34 (35.05)	30 (30.93)	33 (34.02)	

Bold indicates p<0.05.

**Table 2:** Associations of *TOPORS-AS1* with ovarian cancer survival.

Variable	PFS		P value	OS		P value	Adjusted PFS^a^		P value	Adjusted OS^a^		P value
	HR	95% CI		HR	95% CI		HR	95% CI		HR	95% CI	
Low	1			1			1			1		
Mid	0.73	0.47–1.14	0.16	0.85	0.53–1.34	0.48	0.76	0.49–1.17	0.21	0.89	0.55–1.40	0.58
High	0.75	0.49–1.16	0.10	**0.49**	**0.29–0.82**	**0.0069**	0.995	0.64–1.55	0.98	**0.58**	**0.34–0.99**	**0.046**
Continuous	0.87	0.69–1.08	0.21	**0.71**	**0.55–0.91**	**0.0068**	0.99	0.79–1.25	0.94	**0.77**	**0.60–0.999**	**0.049**

*PFS* progression-free survival, *OS* overall survival.

^a^Adjusted for age at surgery, disease stage, tumor grade, and histologic type.

Bold indicates p<0.05.

**Figure 1:**
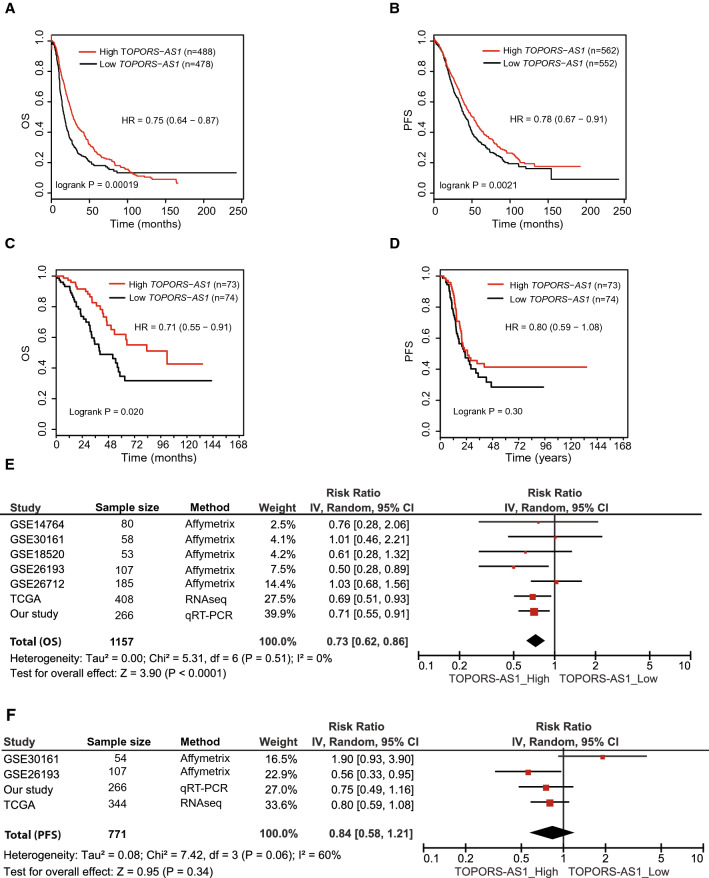
Kaplan–Meier survival curves in ovarian cancer patients with high and low *TOPORS-AS1* expression. (**A**) Kaplan–Meier overall survival curves by high (upper tertile) and low (lower tertile) expression of *TOPORS-AS1* from online Kaplan–Meier Plotter. (**B**) Kaplan–Meier progression-free survival curves by high (upper tertile) and low (lower tertile) expression of *TOPORS-AS1*from online Kaplan–Meier Plotter. (**C**) Kaplan–Meier overall survival curves by high (upper tertile) and low (lower tertile) expression of *TOPORS-AS1* from our study. (**D**) Kaplan–Meier progression-free survival curves by high (upper tertile) and low (lower tertile) expression of *TOPORS-AS1* from our study. (**E**) Forest plots for associations between *TOPORS-AS1* expression and overall survival. (**F**) Forest plots for associations between *TOPORS-AS1* expression and progression-free survival.

Using the Kaplan–Meier Plotter to analyze an online dataset of 1566 patients, we found that *TOPORS-AS1* expression was associated with both overall and progression-free survivals. Patients with high *TOPORS-AS1* had lower risk of death and disease progression compared to those with low expression (Fig. 1A,B), although our study showed a significant association only with overall survival, not progression-free survival (Fig. 1C,D).

Combining our study with 5 GEO (GSE14764, GSE30161, GSE18520, GSE26193, and GSE26712) and one TCGA datasets which contained information on survival outcomes and *TOPORS-AS1* expression in ovarian cancer, we performed a meta-analysis on the association between ovarian cancer survival and *TOPORS-AS1* expression in a total of 1,157 patients. The results showed that patients with high expression of *TOPORS-AS1* had a significantly reduced risk of death, Hazard Ratio (HR) = 0.73, 95% CI 0.55–0.91 (Fig. 1E). Among the 7 datasets, 4 had information on progression-free survival, including GSE30161, GSE26193, TCGA and our study, and the meta-analysis showed no significant association between *TOPORS-AS1* expression and progression-free survival, HR = 0.84, 95% CI 0.58–1.21 (Fig. 1F).

### *TOPORS-AS1* expression in ovarian cancer cells

**Figure 2:**
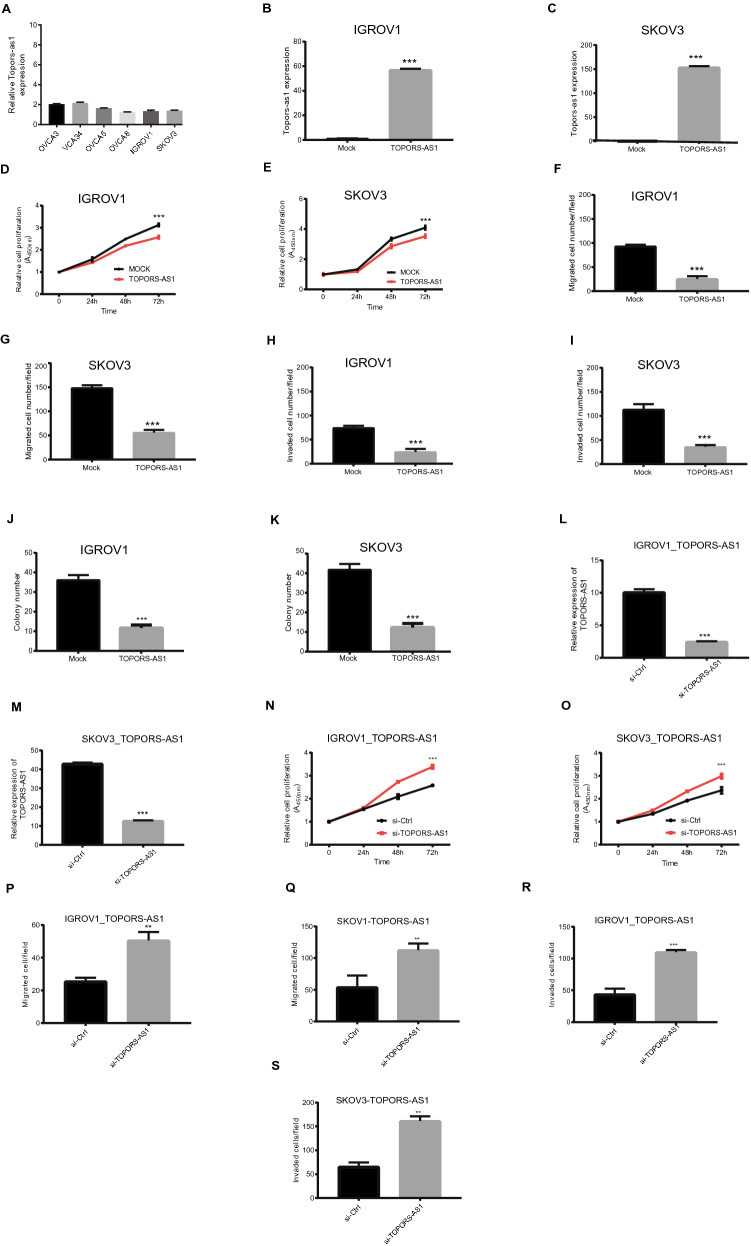
Inhibition of ovarian cancer cell proliferation, invasion, migration, and colony formation by *TOPORS-AS1*. (**A**) RT-qPCR results showing *TOPORS-AS1* expression in 6 ovarian cancer cell lines. (**B**,**C**) RT-qPCR results showing increased *TOPORS-AS1* expression in IGROV1 and SKOV3 after *TOPORS-AS1* transfection. (**D**,**E**) Cell proliferation assay showing slower proliferation of IGROV1 and SKOV3 after *TOPORS-AS1* transfection. (**F**,**G**) Cell transwell migration assay showing reduced cell migration in IGROV1 and SKOV3 after *TOPORS-AS1* transfection. (**H**,**I**) Cell transwell invasion assay showing reduced cell invasion in IGROV1 and SKOV3 after *TOPORS-AS1* transfection. (**J**,**K**) Colony formation assay showing fewer colonies formed in IGROV1 and SKOV3 after *TOPORS-AS1* transfection. (**L**,**M**) RT-qPCR results showing reduced *TOPORS-AS1* expression after *si-TOPORS-AS1* knockdown in IGROV1 and SKOV3 transfected with the *TOPORS-AS1* plasmid. (**N**,**O**) Cell proliferation assay showing increased cell proliferation after *si-TOPORS-AS1* knockdown in IGROV1 and SKOV3 transfected with the *TOPORS-AS1* plasmid. (**P**,**Q**) Cell transwell migration assay showing increased cell migration after *si-TOPORS-AS1* knockdown in IGROV1 and SKOV3 transfected with the *TOPORS-AS1* plasmid. (**R**,**S**) Cell transwell invasion assay showing increased cell invasion after *si-TOPORS-AS1* knockdown in IGROV1 and SKOV3 transfected with the *TOPORS-AS1* plasmid. ***P < 0.0001; **P < 0.001.

*TOPORS-AS1* expression was measured in 6 ovarian cancer cell lines (IGROV1, SKOV3, OVCAR3, OVCAR4, OVCAR5, OVCAR8) with RT-qPCR, and none of the tested cell lines showed high expression of this lncRNA (Fig. 2A). To assess its effect on ovarian cancer cells, we constructed a *TOPORS-AS1* plasmid and transfected two cell lines (IGROV1, SKOV3) to make stable overexpression of the lncRNA*.* Compared to the mock cells, *TOPORS-AS1* expression was about 60-fold higher in the transfected IGROV1 and 150-fold higher in transfected SKOV3 (Fig. 2B,C). We evaluated cell proliferation, migration, invasion, and colony formation in these transfected cells. The experiments showed that *TOPORS-AS1* overexpression led to reduced cell proliferation, migration, invasion, and colony formation (Fig. 2D–K). These results were consistent in both cell lines (Supplemental Figure S1A,B).

To confirm the above effects from *TOPORS-AS1*, we treated the *TOPORS-AS1* overexpressing cells (SKOV3, IGROV1) with siRNAs targeting the lncRNA. After the treatment, *TOPORS-AS1* expression was significantly decreased in the cells (Fig. 2L,M). Compared to the control cells (treated with scrambled siRNAs), cells with *TOPORS-AS1* knockdown had increased proliferation, migration, and invasion (Fig. 2N,S), and the results were consistent in both cell lines (Supplemental Figure S1C).

### Interaction between *TOPORS-AS1* and the Wnt/β-catenin pathway

**Figure 3:**
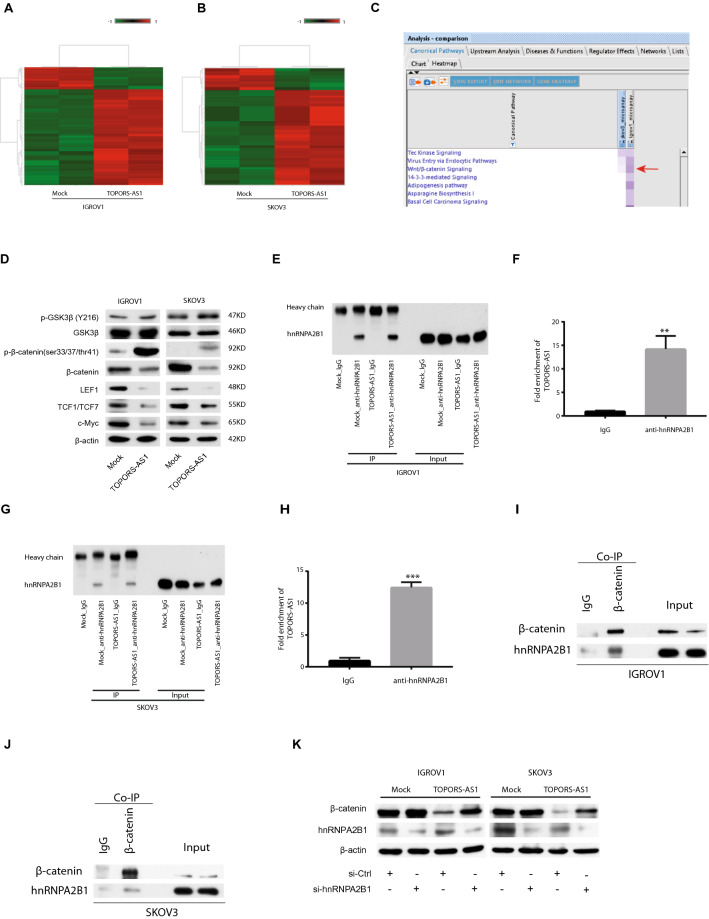
Transcriptomic analysis of ovarian cancer cells associated with *TOPORS-AS1* expression and discovery of β-catenin involvement. (**A**,**B**) Heatmap of differentially expressed genes due to *TOPORS-AS1* overexpression in IGROV1 and SKOV3. (**C**) IPA results of common pathways associated with *TOPORS-AS1* overexpression in both IGROV1 and SKOV3. (**D**) Western blot results showing reduced β-catenin, LEF1, TCF1/TCF7 and c-Myc, increased phosphorylation of β-catenin, and no changes in GSK3β (either total or phosphorylated) in both IGROV1 and SKOV3 due to *TOPORS-AS1* overexpression. (**E**,**G**) Western blot results showing the pulldown of hnRNPA2B1 in mock and *TOPORS-AS1* transfected IGROV1 and SKOV3. (**F**,**H**) RIP results showing marked enrichment of *TOPORS-AS1* in cell extracts processed with anti-hnRNPA2B1 antibody pulldown, but not in IgG pulldown, from IGROV1 and SKOV3. (**I**,**J**) Co-immunoprecipitation (IP) results suggesting β-catenin binding to hnRNPA2B1 in IGROV1 and SKOV3. (**K**) Western blot results showing that β-catenin suppression by *TOPORS-AS1* could be reversed by knockdown hnRNPA2B1 through si-hnRNPA2B1 in IGROV1 and SKOV3. ***P < 0.0001; **P < 0.001.

To investigate the effect of *TOPORS-AS1* in ovarian cancer, we compared the transcriptomes of SKOV3 and IGROV1 with and without *TOPORS-AS1* overexpression using the Human Transcriptome Array 2.0 (Affymetrix). Figure 3A,B are heatmaps of the differentially expressed genes (DEGs) related to *TOPORS-AS1* overexpression in IGROV1 and SKOV3, respectively. There were 51 up-regulated and 6 down-regulated genes (fold change ≥ 1.5 and p < 0.05) which were shared by both cell lines. This list of DEGs was uploaded to IPA for interrogation of the biological networks involving *TOPORS-AS1* overexpression*.* As shown in Fig. 3C, the involvement of Wnt/β-catenin was suggested in both cell lines overexpressing *TOPORS-AS1*.

To determine if the Wnt/β-catenin signaling was involved, we analyzed several major components of the pathway in the transfected cells. Figure 3D shows the results of our western blot analysis in which we found increased phosphorylation of β-catenin and decreased levels of β-catenin, LEF1, TCF1/TCF7, and c-Myc in *TOPORS-AS1* overexpressing cells. GSK3β, both total and phosphorylated form (Y216), showed no changes. As phosphorylation of β-catenin leads to its degradation which results in suppression of the Wnt/β-catenin signaling, our results support the IPA prediction that *TOPORS-AS1* may interrupt the Wnt/β-catenin pathway in ovarian cancer.

To further assess the lncRNA’s effect on β-catenin, we analyzed the β-catenin gene (*CTNNB1*) expression in cells with and without *TOPORS-AS1* overexpression. Our qPCR results showed that levels of β-catenin mRNA were significantly reduced in IGROV1 and SKOV3 overexpressing *TOPORS-AS1*, compared with the mock cells (Supplemental Figure S2), suggesting that *TOPORS-AS1* overexpression may suppress both β-catenin transcription and phosphorylation.

RIP assay was performed to determine if *TOPORS-AS1* interacts with β-catenin in IGROV1 and SKOV3. The analysis showed no direct interaction between these molecules (data not shown), suggesting that the effect of *TOPORS-AS1* on β-catenin may involve or act through other molecules.

### Interaction between *TOPORS-AS1* and hnRNPA2B1

In search for molecules which may interact with *TOPORS-AS1*, we used an online program NPInter^19^ that makes in silico prediction between RNAs and proteins. The software predicted several candidate molecules which may contain putative regions interactive with *TOPORS-AS1*. Among them, hnRNPA2B1 (heterogeneous nuclear ribonucleoproteins A2/B1) was shown to have a binding site in the exon region of *TOPORS-AS1*. This hypothetic interaction was supported by our RIP assay in which hnRNPA2B1 was shown to interact with *TOPORS-AS1* both in IGROV1 and SKOV3 (Fig. 3E–H). Using co-immunoprecipitation, we also found that hnRNPA2B1 could interact with β-catenin, and the interaction was observed in both cell lines (Fig. 3I,J). As mentioned earlier, *TOPORS-AS1* overexpression could reduce the level of β-catenin. When we suppressed the expression of hnRNPA2B1 with siRNA, β-catenin was increased in the cells overexpressing *TOPORS-AS1* (Fig. 3K), suggesting that hnRNPA2B1 may be required in the inhibition of β-catenin by *TOPORS-AS1*.

### Interaction between *TOPORS-AS1* and VDR

**Figure 4:**
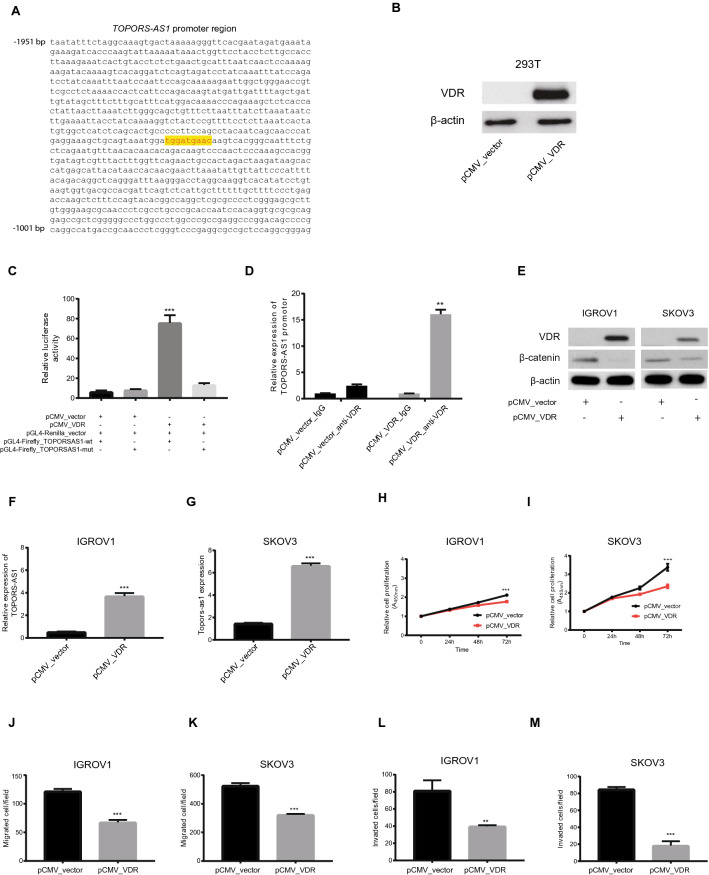
Upregulation of *TOPORS-AS1* expression by VDR and its effect on tumor cell proliferation, migration, and invasion. (**A**) PROMO prediction of a VDR binding site in the *TOPORS-AS1* promoter. (**B**) Western blot results showing increased VDR expression in 293 T after transfecting the cells with a pCMV_VDR plasmid. (**C**) Luciferase reporter assay results showing increased luciferase activity in 293 T cells co-transfected with the VDR plasmid (pCMV_VDR) and luciferase reporter gene linked to *TOPORS-AS1-wt (wild type)* and no increase in *TOPORS-AS1-mut (mutated).* (**D**) Chromatin immunoprecipitation (ChIP) results suggesting VDR binding to the *TOPORS-AS1* promoter. (**E**) Western blot results showing increased VDR and decreased β-catenin in IGROV1 and SKOV3 after *VDR* transfection. (**F**,**G**) RT-qPCR results showing increased *TOPORS-AS1* expression in IGROV1 and SKOV3 after *VDR* transfection. (**H**,**I**) Cell proliferation assay showing slower IGROV1 and SKOV3 proliferation after *VDR* transfection. (**J**,**K**) Cell transwell migration assay showing fewer migrated IGROV1 and SKOV3 after *VDR* transfection. (**L**,**M**) Cell transwell invasion assay showing fewer invaded IGROV1and SKOV3 after *VDR* transfection. ***P < 0.0001; **P < 0.001.

PROMO predicted that vitamin D receptor (VDR) was a transcription factor of *TOPORS-AS1,* and a VDR binding site in the lncRNA’s promoter, from − 1470 to − 1478, was suggested (Fig. 4A). To validate the VDR binding site, we made a VDR vector, pCMV_VDR, which was transfected to 293 T cells. The transfection increased VDR expression in the cells (Fig. 4B), and the increase in VDR expression led to elevated luciferase activities in the cells co-transfected with a wild-type *TOPORS-AS1* promoter, but not in those with a mutant promoter (Fig. 4C). The binding between VDR and the *TOPORS-AS1* promoter was confirmed by chromatin immunoprecipitation (Fig. 4D).

**Figure 5:**
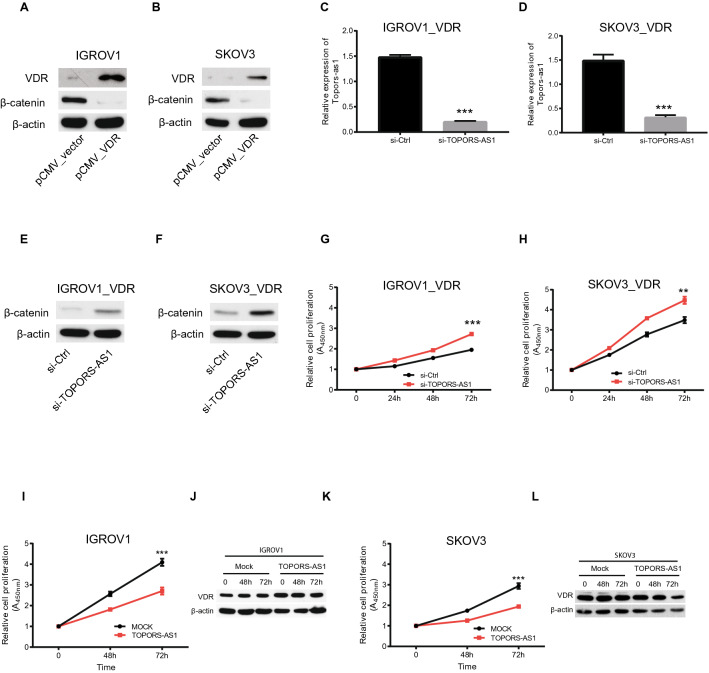
*TOPORS-AS1* mediates the effect of VDR on ovarian cancer cells*.* (**A**,**B**) Western blot results showing reduced β-catenin in IGROV1 and SKOV3 after *VDR* transfection. (**C**,**D**) RT-qPCR results showing *TOPORS-AS1* knockdown by *si-TOPORS-AS1* in *VDR*-transfected IGROV1 and SKOV3. (**E**,**F**) Western blot results showing increased β-catenin after *TOPORS-AS1* knockdown by *si-TOPORS-AS1* in *VDR*-transfected IGROV1 and SKOV3. (**G**,**H**) Increased cell proliferation in *VDR*-transfected IGROV1 and SKOV3 after *TOPORS-AS1* knockdown by *si-TOPORS-AS1.* (**I**,**K**) Decreased cell proliferation in IGROV1 and SKOV3 after transfection with *TOPORS-AS1.* (**J**,**L**) Western blot results showing no change in VDR levels over time in IGROV1 and SKOV3 after transfection with *TOPORS-AS1*. ***P < 0.0001; **P < 0.001.

After confirming the interaction between VDR and *TOPORS-AS1* in 293 T cell, we evaluated the VDR effects on tumor cells by transfecting IGROV1 and SKOV3 with the VDR plasmid. Our experiments showed that the VDR-transfected tumor cells had reduced β-catenin (Fig. 4E) and increased *TOPORS-AS1* expression (Fig. 4F,G). VDR transfection also led to decreased cell proliferation (Fig. 4H,I), migration (Fig. 4J,K), and invasion (Fig. 4L,M). The results were consistent in both cell lines (Supplemental Figure S3). To examine if the VDR effect on β-catenin was mediated through *TOPORS-AS1*, we transfected IGROV1 and SKOV3 first with VDR (Fig. 5A,B) and then *TOPORS-AS1* siRNAs (Fig. 5C,D). After siRNA knockdown, we observed no β-catenin suppression (Fig. 5E,F), and cell proliferation was increased (Fig. 5G,H), suggesting that *TOPORS-AS1*may mediate the VDR effect on tumor cells.

We also evaluated if *TOPORS-AS1* overexpression in ovarian tumor cells could increase the expression of VDR which subsequently suppressed tumor proliferation. Our experiments showed that VDR expression did not change over time in IGROV1 and SKOV3 after *TOPORS-AS1* transfection (Fig. 5J,L), but cell proliferation was suppressed in the transfected cells (Fig. 5I,K), indicating that *TOPORS-AS1* does not affect the expression of VDR and the lncRNA suppresses cell proliferation, not VDR.

## Discussion

**Figure 6:**
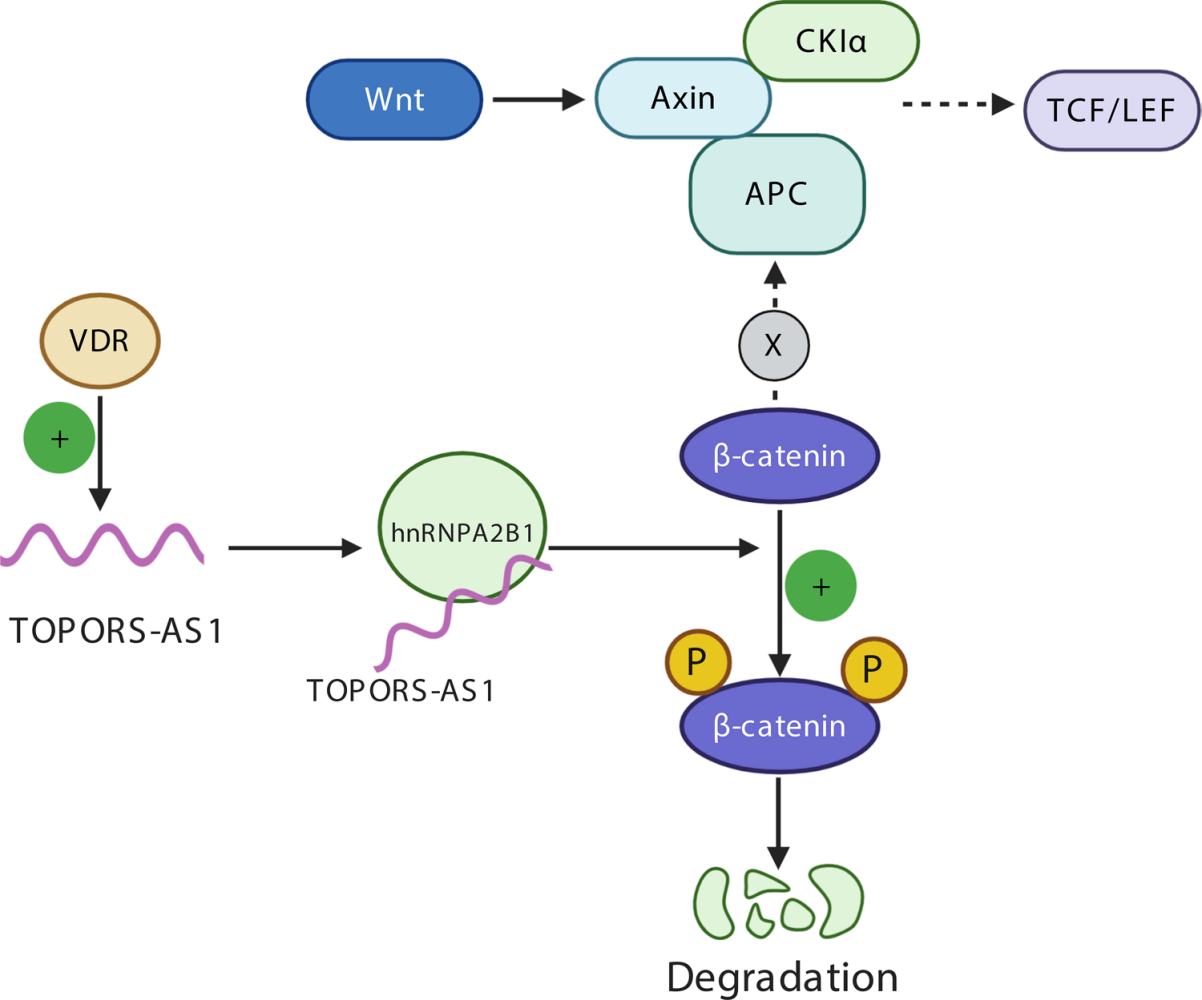
Schematic illustration of *TOPORS-AS1* regulated by VDR, interacting with hnRNPA2B1, and inhibiting the Wnt/β-catenin signaling.

In this study, we found that ovarian cancer patients with early-stage disease tended to have high *TOPORS-AS1* in the tumor and that high expression was associated with favorable overall survival compared to low expression. This survival association was not affected by disease stage and tumor histology and replicated in several independent datasets. A meta-analysis of 1157 patients confirmed the association between *TOPORS-AS1* and overall survival. Results of our in vitro experiments supported the finding in patients, demonstrating that *TOPORS-AS1* expression in ovarian cancer cells inhibited tumor cell proliferation, migration, invasion, and colony formation. Further investigation of biological function and molecular regulation revealed that *TOPORS-AS1* interacted with hnRNPA2B1 and increased the phosphorylation of β-catenin which led to its degradation, resulting in suppression of the Wnt/β-catenin signaling pathway. Our experiments also discovered that VDR was a transcription factor for *TOPORS-AS1* and that the suppression of ovarian cancer cells by VDR could be mediated through *TOPORS-AS1*. The relationships among *TOPORS-AS1*, VDR, β-catenin, and hnRNPA2B1 are depicted in Fig. 6.

*TOPORS-AS1* is encoded by a gene in chromosome 9p21, and the gene has a 554 bp transcript (NR_033992.2). The function and regulation of this transcript was not known before. We found in the study that *TOPORS-AS1* expression was different by disease stage, but not by tumor histology or grade, suggesting that the lncRNA’s expression may be affected by the size or time of tumor growth, but not by the type or differentiation of tumor cells. Our finding of no difference in expression between serous and non-serous tumors also indicates that the biomarker may have broader clinical implications in ovarian cancer, not only to those high-grade serous tumors. Our study showed that *TOPORS-AS1*′s association with survival was only observed consistently in overall survival, but not in progression-free survival. It is unclear what the reasons for this discrepancy are, but we acknowledge the fact that progression-free survival of ovarian cancer is difficult to assess consistently across studies compared to overall survival given its high mortality.

The finding of high *TOPORS-AS1* in association with favorable ovarian cancer survival is in agreement with the observations of two previous studies by Su et al.^20^ and Sorenson et al.^21^ in breast cancer. In addition, our finding of VDR in regulation of *TOPORS-AS1* expression is also in line with the understanding of VDR’s role in cancer^22^. VDR belongs to the superfamily of steroid hormone receptors^23^, and the protein is expressed in many organs including the ovaries where it regulates important cellular activities and biological functions^15,24^. VDR is known to be a strong tumor suppressor and is able to inhibit ovarian cancer cell growth^15^. We found in the study that VDR was able to bind to the *TOPORS-AS1* promoter, upregulating its expression. Our experiments also indicated that *TOPORS-AS1* could mediate the inhibitory effects of VDR on cell proliferation, migration, and invasion.

Our study further suggests that *TOPORS-AS1* may play a role in connection to the reciprocal suppression between VDR and the Wnt/β-catenin pathway in ovarian cancer^25^. It is known that VDR deficiency leads to increased Wnt/β-catenin signaling in colon cancer^26^. When the pathway is activated, β-catenin is translocated from cytoplasm to nucleus where it binds to LEF/TCF to increase the expression of Myc and Cyclin D1, promoting cell migration and tumor progression. When deactivated, β-catenin is released from the APC/Axin/GSK3β complex in cytoplasm, undergoing phosphorylation and degradation^27^. In various human malignancies including ovarian cancer, the Wnt/β-catenin signaling is highly activated^28,29^. Defects in APC or β-catenin were found in colorectal cancer, which stabilized β-catenin, resulting in constitutive activation of the Wnt/β-catenin pathway^30–32^. Evidence suggests that the Wnt/β-catenin pathway is also important in ovarian cancer^28,33–35^.

A number of lncRNAs have been identified to influence or regulate the Wnt/β-catenin pathway^36–41^. In our experiments, we found increased *TOPORS-AS1* expression in ovarian cancer cells after VDR transfection, and these increases in expression of *TOPORS-AS1* and VDR resulted in decreased β-catenin expression and increased phosphorylation of β-catenin, leading to suppression of the Wnt/β-catenin pathway. To evaluate which one, VDR or *TOPORS-AS1*, was responsible for the change in β-catenin, we added si-*TOPORS-AS1* in cell culture and found that the decline in β-catenin was abolished after *TOPORS-AS1* knockdown, suggesting that the lncRNA may mediate the effect of VDR on β-catenin. Our experiments further indicated no direct interaction between *TOPORS-AS1* and β-catenin, and the effect of *TOPORS-AS1* on β-catenin involved an RNA-binding protein. We found that hnRNPA2B1 was able to interact with both *TOPORS-AS1* and β-catenin. This protein is a member of the heterogeneous nuclear ribonucleoprotein family which functions as a splicing factor to process and stabilize pre-mRNAs. *MYU* (c-Myc-upregulated lncRNA) associates with the RNA binding protein hnRNP-K to stabilize CDK6 expression, and the lncRNA is a direct target of the Wnt/c-Myc pathway^5^. It was found that hnRNPA2 could interact with the 3′-UTR of *CTNNB1*, affecting the expression of *CTNNB1* and β-catenin^42^. In our study, β-catenin levels were decreased by *TOPORS-AS1* through its interaction with hnRNPA2B1. Without the RNA-binding protein, the effect of *TOPORS-AS1* on β-catenin was not observed, suggesting that both *TOPORS-AS1* and hnRNPA2B1 are required in their action on β-catenin.

In summary, our study showed that high expression of lncRNA *TOPORS-AS1* was associated with favorable prognosis of ovarian cancer. In vitro experiments demonstrated that *TOPORS-AS1* behaved like a tumor suppressor in ovarian cancer as the lncRNA could suppress cell proliferation, migration, invasion, and colony formation. Furthermore, *TOPORS-AS1* increased β-catenin degradation and inhibited the expression of *CTNNB1*, suppressing the Wnt/β-catenin signaling. Our experiments also found that VDR was able to bind to the *TOPORS-AS1* promoter, upregulating its expression, and that *TOPORS-AS1* could mediate the inhibitory effects of VDR on cell proliferation, migration, and invasion. The effect of *TOPORS-*AS1 on β-catenin in ovarian cancer relied on the presence of hnRNPA2B1. Taken together, these findings suggest that *TOPORS-AS1* is a tumor suppressor in ovarian cancer and that assessing the lncRNA level in tumor tissue may help to predict the disease prognosis and design treatment strategy.

## Materials and methods

### Study patients

To study epithelial ovarian cancer, we recruited 266 patients who were operated in two hospitals affiliated with the University of Turin in Italy. The recruitment was approved by the AOU Citta della Salute’s ethic review committee and the Mauriziano hospital’s eithic review committee, and informed consents were obtained from the patients. All the study protocols were performed in accordance with relevant guidelines and regulations. Disease stage was determined based on the International Federation of Gynecology and Obstetrics (FIGO) Classification, and tumor histology and grade were characterized according to the WHO Guidelines^43,44^. Detailed description of the study patients is provided in Supplemental Materials and Methods S1.

### Tumor samples and RNA extraction

Fresh tumor samples were collected from the patients during surgery, and the samples were evaluated independently by two pathologists to confirm tumor cell contents (> 80% tumor cells). The tissue specimens were pulverized using a tissue homogenizer. Approximately 30 mg of pulverized tissue powder was used for total RNA extraction, using the AllPrep DNA/RNA Mini kit (Qiagen). After extraction, total RNAs were treated with RNase-free DNase to remove DNA contamination. The purity and quantity of the RNA samples were evaluated with specific light absorbances and RNA Integrity Number (RIN). The same methods were also used for RNA extraction and quantification from cell lines.

### RT-qPCR

A high capacity cDNA reverse transcription kit was used to convert total RNA to cDNA (Applied Biosystems). The cDNA samples were analyzed for expression of *TOPORS-AS1, CTNNB1 and GAPDH,* using the SYBR Green-based quantitative PCR. PCR primer sequences are shown in Supplemental Table S1. Details of the analysis are provided in Supplemental Materials and Methods S1.

### Cell culture

The NCI-60 DTP Human Tumor Cell Screening Panel was purchased from NIH for research by the High-throughput Drug Screening Core at University of Hawaii Cancer Center (UHCC). From the panel we selected six ovarian cancer cell lines for testing, including OVCAR3, OVCAR4, OVCAR5, OVCAR8, IGROV1, and SKOV3. The 293 T cell line was purchased from the American Type Culture Collection (ATCC). All the cells were cultured in RPMI 1640 medium containing 10% FBS and 100 units/ml of penicillin/streptomycin (Pen/Strep) at 37 °C under a humidified atmosphere containing 5% CO_2_.

### Plasmid construction and amplification

Based on the transcript NR_033992.2 (554 bp), a *TOPORS-AS1* template was synthesized by GENEWIZ and inserted into a lentiviral vector, pCDH-EF1-MCS-pA-PGK-copGFP-T2A-Puro (System Biosciences), with two restriction sites, Nhe1 and BamH1. The plasmid contains two promoters, EF1 which controls the expression of *TOPORS-AS1* and PGK which drives a report gene, GFP, serving as a transfection control. The expression of *TOPORS-AS1* from the plasmid was verified by direct sequencing and gel electrophoresis after restriction enzyme digestion. For plasmid amplification, please see details in Supplemental Materials and Methods S1.

### Plasmid transfection and stable cell selection

Ovarian cancer cell lines, IGROV1 and SKOV3, were selected for transfection either with pCDH_*TOPORS-AS1* or pCDH_vector (mock), using the Lipofectamine 3000 reagent (Thermo Fisher Scientific). Details of plasmid transfection and cell selection are provided in Supplemental Materials and Methods S1.

### Cell proliferation assay

Ovarian cancer cells in 100 μl complete medium were added into the wells of 96-well plates at a density of 3 × 10^3^ cells per well. After 0, 24, 48, and 72 h of incubation, 10 μl cell proliferation reagent WST-1 was added into each well following the manufacturer’s instructions (Roche). The cells were incubated with WST-1 for 2 more hours at 37 °C in a humidified atmosphere containing 5% CO_2_ before the plate was read with a microplate reader (BioTek Synergy 2, Winooski, VT) at 450 nm absorbance. Cell proliferation at each time point was normalized to the mean viable cells at 0 h. Each proliferation assay was performed in triplicate.

### Cell migration and invasion assays

Cell migration and invasion were analyzed in 24-well plates using the Costar Transwell permeable membrane support with 8.0-μm pore size (Corning). In the invasion assay, ovarian cancer cells, 1 × 10^4^ cells per well, in 200 μl serum-free medium were placed in the upper chambers coated with growth-factor-reduced Matrigel at 1 mg/ml (BD Pharmingen). The lower chambers were filled with 600 μl complete culture media containing 10% FBS. Cell migration assays were performed similarly without Matrigel coating. Invaded or migrated cells were stained with HEME 3 Solution (Fisher Diagnostics) after 36 h of culture, and the stained cells were photographed using the Olympus CKX41 microscopy with an Infinity 2 camera. Cell numbers were counted using the ImageJ software. All the experiments were repeated 3 times, and each experiment was done in triplicate.

### Colony formation assay

The assay has been described elsewhere^8^. In brief, ovarian cancer cells (5 × 10^3^) were seeded on 0.3% agarose overlaid onto solidified 0.6% agarose in PRMI 1640 with 10% FBS. Complete medium (200 µl) was added into the well every three days. Cell colonies were counted after three weeks. The experiments were repeated 3 times.

### siRNA assay

Ovarian cancer cells with stable expression of *TOPORS-AS1* were transfected with the Target Long Noncoding RNA siRNA-SMART pool (#R-189076-00-0005) to knockdown *TOPORS-AS1* expression, and Lincode Non-targeting Pool (#D-001320-10-05) was used as a negative control, both of which were purchased from Dharmacon. The transfection was performed following the manufacturer’s protocol for Lipofectamine RNAiMAX (Themo Fisher Scientific). The transfected cells were prepared for analysis after 36 h of incubation.

### Analysis of cell transcriptome

The transcriptomes of IGROV1 and SKOV3 with and without *TOPORS-AS1* overexpression were analyzed with the Affymetrix Human Transcriptome Array 2.0 (Affymetrix). DNA labeling, probe hybridization, and signal scanning were completed by the Genomics Shared Resource at UHCC. The initial array data were normalized using the robust multi-array average RMA algorithm in the Affymetrix Expression Console (Affymetrix). Gene-based differential expression were analyzed using the Transcriptome Analysis Console (TAC) v3.0 (Affymetrix). Differentially expressed genes, defined as fold change ≥ 1.5 and p value < 0.05, were interrogated with the Ingenuity Pathway Analysis (IPA) software to predict the possible signaling pathways and regulatory mechanisms associated with *TOPORS-AS1* overexpression. PROMO (http://alggen.lsi.upc.es/cgi-bin/promo_v3/promo/promoinit.cgi?dirDB=TF_8.3) was employed with 5% dissimilarity to predict the potential transcription factors and their possible binding sites in the *TOPORS-AS1* promoter^45,46^. NPInter (https://www.bioinfo.org/NPInter/) was used to show possible interactions between *TOPORS-AS1* and other molecules^19^.

### RNA immunoprecipitation (RIP)

EZ-Magna RIP RNA-Binding Protein Immunoprecipitation kit (#17-701, Millipore) was used for RIP assay. Antibody against β-catenin (ab6302) was purchased from Abcam. Rabbit IgG antibody (#12-370 from EMD Millipore) was used as control. RIP assay was performed according to the manufacturer's instructions.

### Chromatin immunoprecipitation (ChIP)

ChIP assay was carried out using a commercial kit from EMD Millipore. Plasmids (pCMV-vector or pCMV-VDR) were transfected to 293 T cells using the Lipofectamine 3000 reagent (Themo Fisher Scientific). Details of the ChIP assay are described in Supplemental Materials and Methods S1.

### Dual luciferase reporter assay

A plasmid (pCMV-VDR) containing a full-length human *VDR* and a vector (pGL4.27[luc2P/minP/Hygro]) were purchased from Origene Technologies and Promega, respectively. GENEWIZ synthesized wild and mutant *TOPORS-AS1* promoters and inserted the sequences into the pGL4.27 vector to make pGL4.27-*TOPORS-AS1*-wt and pGL4.27-*TOPORS-AS1*-mut plasmids. pCMV-VDR or pCMV-vector were transiently transfected to the 293 T cells using Lipofectamine 3000 reagent (Themo Fisher Scientific). After transfection, cells were incubated for 36 h and further transfected with plasmid pGL4.27-*TOPORS-AS1*-wt or pGL4.27-*TOPORS-AS1*-mut, together with the renilla reporter vector. After incubation, renilla and firefly luciferase activities were measured using the Dual-Luciferase kit (Promega). The results were normalized to the renilla reporter to adjust for transfection efficiency. Each assay was performed in triplicate, and the assays were repeated three times.

### Immunoblotting

Cell proteins were extracted using the RIPA Lysis and Extraction Buffer (Themo Fisher Scientific). Concentrations of the extracted proteins were measured with the BCA assay, and 40–60 µg of the proteins were boiled in the Laemmli Sample Buffer (Bio-Rad) at 95℃ for 5 min, followed by 10% SDS–PAGE. The gel results were transferred onto the polyvinylidene difluoride (PVDF) membranes (Millipore) which were then blocked with 5% non-fat milk for 30 min and incubated with a primary antibody, followed by a secondary antibody to generate the signals detectable by an enhanced chemiluminescence system (Pierce). Primary antibodies used for blotting included anti-VDR (#12550 from Cell Signaling Technology), anti-hnRNPA2B1 (ab6102 from Abcam), anti-β-catenin (ab6302 from Abcam), anti-p-β-catenin (ser33/37/thr41) (#9561 from Cell Signaling Technology), anti-β-catenin (#8480 from Cell Signaling Technology), anti-LEF1 (#2230 from Cell Signaling Technology), anti-TCF1/TCF7(#2203 from Cell Signaling Technology), anti-cMYC (#18583 from Cell Signaling Technology), anti-p-GSK3β(Y216) (ab75745 from Abcam), anti-GSK3β (#12456 from Cell Signaling Technology, and anti-β-actin (A2228 from Sigma-Aldrich) antibodies.

### Immunoprecipitation (IP)

Cells were cultured to 70% confluence and lysed in the Pierce IP Buffer (Themo Fisher Scientific). To purify proteins, cell lysates (600–800 μg) were incubated with 30 μl of the Pierce Protein A/G Magnetic Beads (Themo Fisher Scientific) for 30 min at 4 °C on rotation. After bead removal and overnight incubation at 4 °C with anti-β-catenin antibody (2–4 μg/mg protein), the protein solution was mixed with 30 μl of the Protein A/G Magnetic Beads, and the mixture was incubated for another 2 h. Then, the beads were washed three times with washing buffer and once with PBS, and were further mixed with the Laemmli Sample Buffer, followed by boiling at 95 °C for 5 min. After that, the solution was processed with 10% SDS–PAGE, and the resulting gels were transferred to the PVDF membranes, followed by incubation with another antibody (anti-hnRNPA2B1) and ECL measurement (Bio-Rad).

### Meta-analysis

Using the keywords “ovarian cancer” and “ovarian carcinoma”, we searched the Gene Expression Omnibus (GEO) in NCBI (https://www.ncbi.nlm.nih.gov/gds). Datasets with more than 50 tumor samples as well as information on *TOPORS-AS1* expression and survival outcomes were selected for analysis. Five datasets meeting the criteria were identified in the database, including GSE14764, GSE30161, GSE18520, GSE26193, and GSE26712. The Cancer Genome Atlas (TCGA) data on *TOPORS-AS1* expression in ovarian cancer were also downloaded from TANRIC (https://www.tanric.org), and clinical information related to the dataset was retrieved from cBioPortal (http://www.cbioportal.org)^47,48^. Hazard ratios (HR) and 95% confidence intervals (95% CI) were calculated for each of the datasets, and these results were entered into the Review Manager 5.3 to obtain summary HR and 95% CI, using the random-effect model^49^.

### Statistical analysis

Expression index (EI) was calculated as levels of *TOPORS-AS1* expression after adjusting for the *GAPDH* expression, and the calculation was based on the formula 1000 × 2^(−∆Ct)^, where ∆Ct is the difference in cycle thresholds (Ct) between Ct_*TOPORS-AS1*_ and Ct_*GAPDH*_. For data analysis, the EI values were classified into low, middle, and high groups, based on the tertile distribution of *TOPORS-AS1* expression. The expression data were analyzed for associations with clinical and pathological variables, using the chi-square test. Log-rank test was used to compare the Kaplan–Meier survival curves between high and low expression of *TOPORS-AS1*, and the median was used as cutoff. Hazard ratios (HR) and 95% confidence interval (CI) were calculated using the Cox proportional hazards regression model, univariate and multivariate. In the multivariate model, age at surgery, disease stage, tumor grade and histology type were included as covariates to control for confounding factors. Two survival outcomes, progression-free and overall survivals, were used in survival analysis. Progression-free survival was the time interval from the date of surgery to the date of disease progression or last follow-up. Overall survival was the duration between surgery and last follow-up or death. SAS (version 9.4) and R (version 3.0.2) were used for statistical analyses. All p-values were two-sided.

## Supplementary Information

Supplementary Information

## Abbreviations

lncRNAs: Long non-coding RNAs
TOPORS-AS1: TOPORS Antisense RNA
VDR: Vitamin D receptor
MEG3: Maternally expressed gene 3
HR: Hazard ratio
CI: Confidence interval
OS: Overall survival
PFS: Progression free survival
DEGs: Differentially expressed genes
LEF1: Lymphoid enhancer-binding factor 1
TCF1/TCF7: Transcription Factor 7
GSK3β: Glycogen synthase kinase 3 beta
hnRNPA2B1: Heterogeneous nuclear ribonucleoproteins A2/B1
IP: Immunoprecipitation
RIP: RNA immunoprecipitation chip
ChIP: Chromatin immunoprecipitation

## References

[1] ACS. American Cancer Society. Cancer Facts & Figures 2018. *American Cancer Society* **2018** (2018).

[2] Harrow J (2012). GENCODE: The reference human genome annotation for The ENCODE Project. Genome Res..

[3] Gao Y (2015). LncRNA-HOST2 regulates cell biological behaviors in epithelial ovarian cancer through a mechanism involving microRNA let-7b. Hum. Mol. Genet..

[4] Ji Q (2014). Long non-coding RNA MALAT1 promotes tumour growth and metastasis in colorectal cancer through binding to SFPQ and releasing oncogene PTBP2 from SFPQ/PTBP2 complex. Br. J. Cancer.

[5] Kawasaki Y (2016). MYU, a Target lncRNA for Wnt/c-Myc signaling, mediates induction of CDK6 to promote cell cycle progression. Cell. Rep..

[6] Kim J (2018). Long noncoding RNA MALAT1 suppresses breast cancer metastasis. Nat. Genet..

[7] Luo M (2013). Long non-coding RNA H19 increases bladder cancer metastasis by associating with EZH2 and inhibiting E-cadherin expression. Cancer Lett..

[8] Wang Z (2019). ERalpha upregulates the expression of long non-coding RNA LINC00472 which suppresses the phosphorylation of NF-kappaB in breast cancer. Breast Cancer Res. Treat..

[9] Fu Y (2016). Long non-coding RNAs, ASAP1-IT1, FAM215A, and LINC00472, in epithelial ovarian cancer. Gynecol. Oncol..

[10] Carlberg C, Campbell MJ (2013). Vitamin D receptor signaling mechanisms: Integrated actions of a well-defined transcription factor. Steroids.

[11] Feldman D, Krishnan AV, Swami S, Giovannucci E, Feldman BJ (2014). The role of vitamin D in reducing cancer risk and progression. Nat. Rev. Cancer.

[12] Villena-Heinsen C (2002). Immunohistochemical analysis of 1,25-dihydroxyvitamin-D3-receptors, estrogen and progesterone receptors and Ki-67 in ovarian carcinoma. Anticancer Res..

[13] Irani, M. & Merhi, Z. Role of vitamin D in ovarian physiology and its implication in reproduction: A systematic review. *Fertil. Steril.* **102**, 460–468. 10.1016/j.fertnstert.2014.04.046 (2014). 10.1016/j.fertnstert.2014.04.046 24933120

[14] Nandi A, Sinha N, Ong E, Sonmez H, Poretsky L (2016). Is there a role for vitamin D in human reproduction?. Horm. Mol. Biol. Clin. Investig..

[15] Ahonen MH, Zhuang YH, Aine R, Ylikomi T, Tuohimaa P (2000). Androgen receptor and vitamin D receptor in human ovarian cancer: growth stimulation and inhibition by ligands. Int. J. Cancer.

[16] Jiang YJ, Bikle DD (2014). LncRNA profiling reveals new mechanism for VDR protection against skin cancer formation. J. Steroid. Biochem. Mol. Biol..

[17] Xiu YL (2017). Upregulation of the lncRNA Meg3 induces autophagy to inhibit tumorigenesis and progression of epithelial ovarian carcinoma by regulating activity of ATG3. Oncotarget.

[18] Zuo S, Wu L, Wang Y, Yuan X (2020). Long non-coding RNA MEG3 activated by vitamin D suppresses glycolysis in colorectal cancer via promoting c-Myc degradation. Front. Oncol..

[19] Hao, Y. *et al.* NPInter v3.0: an upgraded database of noncoding RNA-associated interactions. *Database (Oxford)*. 10.1093/database/baw057 (2016). 10.1093/database/baw057 PMC4834207 27087310

[20] Su X (2014). Comprehensive analysis of long non-coding RNAs in human breast cancer clinical subtypes. Oncotarget.

[21] Sorensen KP (2015). Long non-coding RNA expression profiles predict metastasis in lymph node-negative breast cancer independently of traditional prognostic markers. Breast Cancer Res..

[22] Deuster, E., Jeschke, U., Ye, Y., Mahner, S. & Czogalla, B. Vitamin D and VDR in gynecological cancers-a systematic review. *Int. J. Mol. Sci.* **18**. 10.3390/ijms18112328 (2017). 10.3390/ijms18112328 PMC5713297 29113037

[23] Whitfield GK, Jurutka PW, Haussler CA, Haussler MR (1999). Steroid hormone receptors: evolution, ligands, and molecular basis of biologic function. J. Cell. Biochem. Suppl..

[24] Silvagno F (2010). Analysis of vitamin D receptor expression and clinical correlations in patients with ovarian cancer. Gynecol. Oncol..

[25] Hu L, Bikle DD, Oda Y (2014). Reciprocal role of vitamin D receptor on beta-catenin regulated keratinocyte proliferation and differentiation. J. Steroid. Biochem. Mol. Biol..

[26] Larriba MJ (2011). Vitamin D receptor deficiency enhances Wnt/beta-catenin signaling and tumor burden in colon cancer. PLoS ONE.

[27] MacDonald BT, Tamai K, He X (2009). Wnt/beta-catenin signaling: Components, mechanisms, and diseases. Dev. Cell.

[28] Boyer A, Goff AK, Boerboom D (2010). WNT signaling in ovarian follicle biology and tumorigenesis. Trends Endocrinol. Metab..

[29] Arend RC, Londono-Joshi AI, Straughn JM, Buchsbaum DJ (2013). The Wnt/beta-catenin pathway in ovarian cancer: A review. Gynecol. Oncol..

[30] Anastas JN, Moon RT (2013). WNT signalling pathways as therapeutic targets in cancer. Nat. Rev. Cancer.

[31] Kinzler KW, Vogelstein B (1996). Lessons from hereditary colorectal cancer. Cell.

[32] Polakis P (2012). Drugging Wnt signalling in cancer. EMBO J..

[33] Gatcliffe TA, Monk BJ, Planutis K, Holcombe RF (2008). Wnt signaling in ovarian tumorigenesis. Int. J. Gynecol. Cancer.

[34] Rask K (2003). Wnt-signalling pathway in ovarian epithelial tumours: Increased expression of beta-catenin and GSK3beta. Br. J. Cancer.

[35] Wend P, Holland JD, Ziebold U, Birchmeier W (2010). Wnt signaling in stem and cancer stem cells. Semin. Cell Dev. Biol..

[36] Ong MS (2017). 'Lnc'-ing Wnt in female reproductive cancers: Therapeutic potential of long non-coding RNAs in Wnt signalling. Br. J. Pharmacol..

[37] Shen, P., Pichler, M., Chen, M., Calin, G. A. & Ling, H. To Wnt or lose: the missing non-coding linc in colorectal cancer. *Int. J. Mol. Sci.* **18**. 10.3390/ijms18092003 (2017). 10.3390/ijms18092003 PMC5618652 28930145

[38] Hu P (2015). NBAT1 suppresses breast cancer metastasis by regulating DKK1 via PRC2. Oncotarget.

[39] Ohtsuka M (2016). H19 noncoding RNA, an independent prognostic factor, regulates essential Rb-E2F and CDK8-beta-catenin signaling in colorectal cancer. EBioMedicine.

[40] Di Cecilia S (2016). RBM5-AS1 is critical for self-renewal of colon cancer stem-like cells. Cancer Res..

[41] Fang C (2017). Long non-coding RNA HNF1A-AS1 mediated repression of miR-34a/SIRT1/p53 feedback loop promotes the metastatic progression of colon cancer by functioning as a competing endogenous RNA. Cancer Lett..

[42] Stockley J (2014). The RNA-binding protein hnRNPA2 regulates beta-catenin protein expression and is overexpressed in prostate cancer. RNA Biol..

[43] Scully RE, Sobin LH (1987). Histologic typing of ovarian tumors. Arch. Pathol. Lab. Med..

[44] Shepherd JH (1989). Revised FIGO staging for gynaecological cancer. Br. J. Obstet. Gynaecol..

[45] Messeguer X (2002). PROMO: Detection of known transcription regulatory elements using species-tailored searches. Bioinformatics.

[46] Farre D (2003). Identification of patterns in biological sequences at the ALGGEN server: PROMO and MALGEN. Nucleic Acids Res..

[47] Gao, J. *et al.* Integrative analysis of complex cancer genomics and clinical profiles using the cBioPortal. *Sci. Signal* **6**, pl1. 10.1126/scisignal.2004088 (2013). 10.1126/scisignal.2004088 PMC4160307 23550210

[48] Cerami E (2012). The cBio cancer genomics portal: an open platform for exploring multidimensional cancer genomics data. Cancer Discov..

[49] DerSimonian R, Laird N (1986). Meta-analysis in clinical trials. Control Clin. Trials.

