# Supplementary material for: Vitamin D receptor upregulates lncRNA TOPORS-AS1 which inhibits the Wnt/β-catenin pathway and associates with favorable prognosis of ovarian cancer
Source: Sci Rep. 2021 Apr 5;11:7484. doi: 10.1038/s41598-021-86923-7 (PMC8021562; doi:10.1038/s41598-021-86923-7)
Supplement: Supplementary file 1 — Supplementary Information [file 41598_2021_86923_MOESM1_ESM.pdf]

Vitamin D receptor upregulates lncRNA *TOPORS-AS1* which inhibits the Wnt/β-catenin pathway and associates with favorable prognosis of ovarian cancer

Short title: *TOPORS-AS1,* β-catenin, and VDR in ovarian cancer

Yuanyuan Fu[1, 4], Dionyssios Katsaros[2], Nicoletta Biglia[3], Zhanwei Wang[1], Ian Pagano[1], Marcus Tius[1], Maarit Tiirikainen[1], Charles Rosser[5], Haining Yang[1], Herbert Yu[1, *]

Figure S1

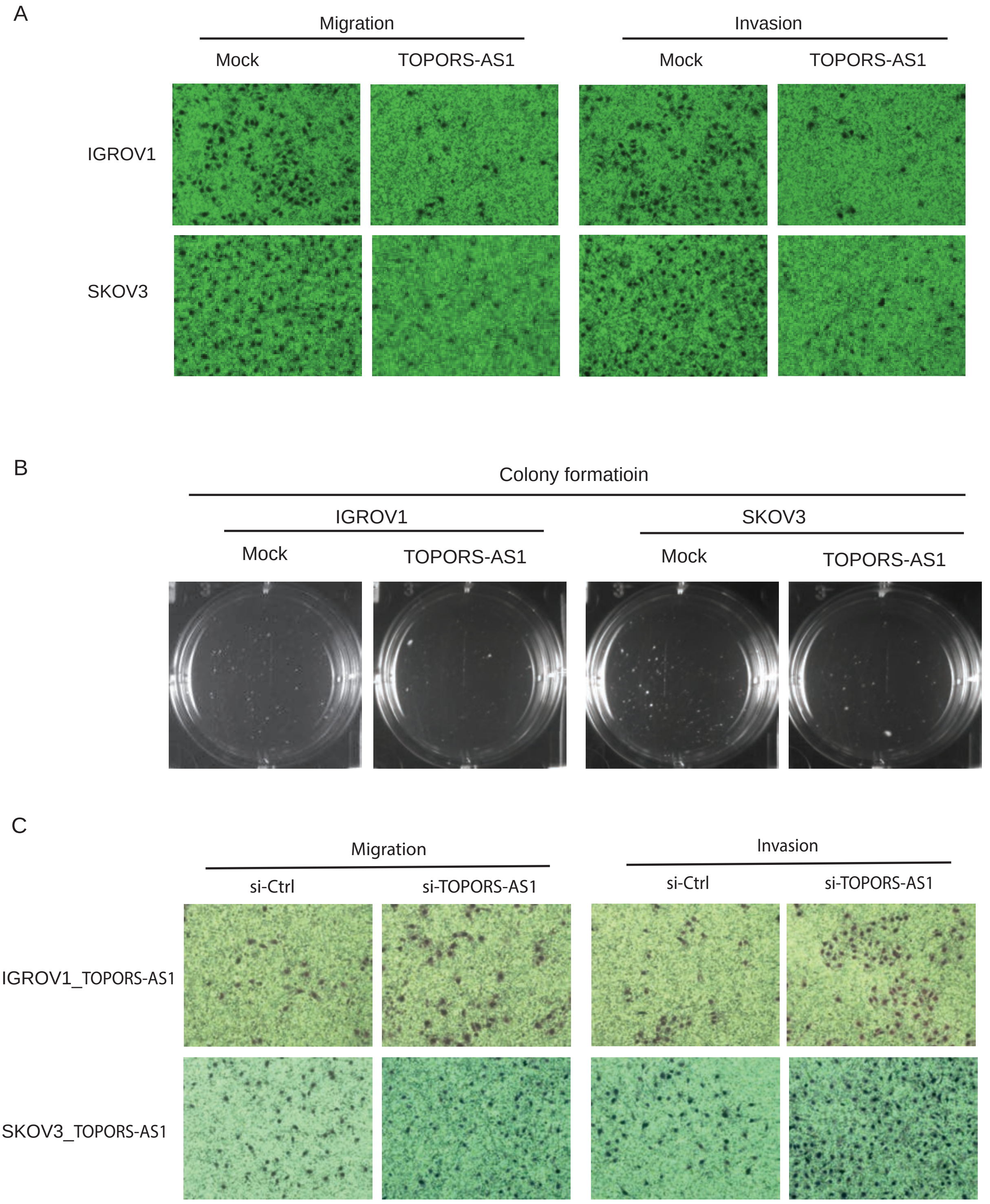
A
Migration
Invasion
Mock
TOPORS-AS1
Mock
TOPORS-AS1
IGROV1
SKOV3
B
Colony formatioin
IGROV1
SKOV3
Mock
TOPORS-AS1
Mock
TOPORS-AS1
C
Migration
Invasion
si-Ctrl
si-TOPORS-AS1
si-Ctrl
si-TOPORS-AS1
IGROV1_TOPORS-AS1
SKOV3_TOPORS-AS1

Figure S2

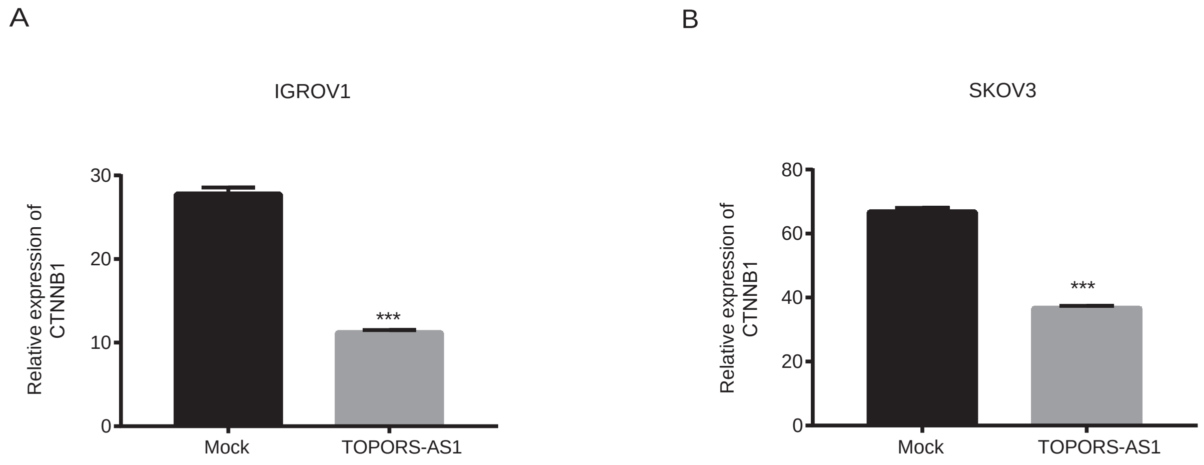

A
IGROV1
Relative expression of CTNNB1
30
20
10
0
***
Mock
TOPORS-AS1
B
SKOV3
Relative expression of CTNNB1
80
60
40
20
0
***
Mock
TOPORS-AS1

Figure S3

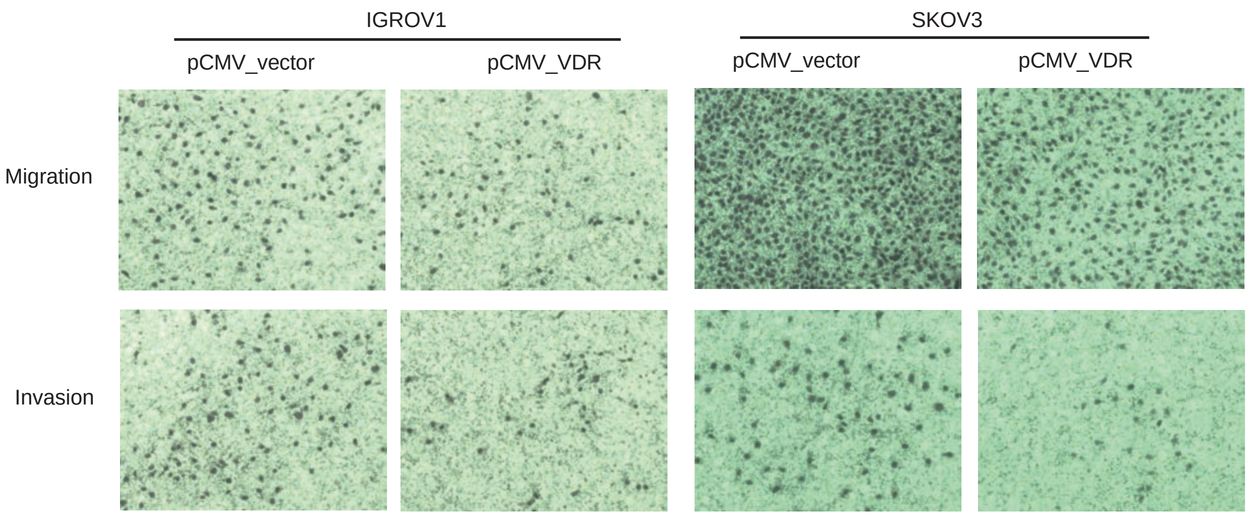
IGROV1
SKOV3
pCMV_vector
pCMV_VDR
pCMV_vector
pCMV_VDR
Migration
Invasion

Vitamin D receptor upregulates lncRNA *TOPORS-AS1* which inhibits the Wnt/β-catenin pathway and associates with favorable prognosis of ovarian cancer

Short title: *TOPORS-AS1*, β-catenin, and VDR in ovarian cancer

Yuanyuan Fu[1, 4], Dionyssios Katsaros[2], Nicoletta Biglia[3], Zhanwei Wang[1], Ian Pagano[1], Marcus Tius[1], Maarit Tiirikainen[1], Charles Rosser[5], Haining Yang[1], Herbert Yu[1, *]

Supplemental Table S1. PCR primer sequences

TOPORS-AS1	Forward	CTGGGAGGTTACTGTAAGGC (Sense)
	Reverse	GCAAGCAGCAACACTGAATAA (Antisense)
TOPORS-AS1 promoter	Forward	GCCTACAATCAGCAACCCAT (Sense)
	Reverse	CGACTATCACCGTGGCTTTG (Antisense)
CTNNB1	Forward	CTTCACCTGACAGATCCAAGTC (Sense)
	Reverse	CCTTCCATCCCTTCCTGTTTAG (Antisense)
GAPDH	Forward	GTCAAGGCTGAGAACGGGAA (Sense)
	Reverse	AAATGAGCCCCAGCCTTCTC (Antisense)

Vitamin D receptor upregulates lncRNA *TOPORS-AS1* which inhibits the Wnt/β-catenin pathway and associates with favorable prognosis of ovarian cancer

Short title: *TOPORS-AS1*, β-catenin, and VDR in ovarian cancer

Yuanyuan Fu[1, 4], Dionyssios Katsaros[2], Nicoletta Biglia[3], Zhanwei Wang[1], Ian Pagano[1], Marcus Tius[1], Maarit Tiirikainen[1], Charles Rosser[5], Haining Yang[1], Herbert Yu[1, *]

Supplemental Materials and Methods

Patient information

The patients were enrolled in the study consecutively, including 191 patients from the University Hospital between October 1991 and February 2000 and 75 patients from Mauriziano Hospital between April 1997 and January 2013. These patients were followed from the time of surgery through June 2005 and March 2015, respectively. The median follow-up time was 29.9 months (range from 0.6 to 114.1 months) for the first group and 40.5 months (range from 3.4 to 165.5 months) for the second one. Patient clinical and pathological information was extracted from medical records and pathology reports. All patients enrolled in the study underwent cytoreduction surgery for primary ovarian cancer, and their tumor samples were collected during surgery. The specimens were snap-frozen in liquid nitrogen immediately after resection and then transferred to -80℃ freezers for storage. Two hundred and eight patients (78%) received standard post-operative platinum-based chemotherapy, which included cisplatin and cyclophosphamide administered between 1991 and 1995 (n=63) and carboplatin and paclitaxel after 1995 (n=145).

RT-qPCR

RNA was converted to cDNA using a cDNA reverse transcription kit (Applied Biosystems). The cDNA samples were analyzed with qPCR for *TOPORS-AS1* expression in the LightCycler 480 instrument (Roche) using the LightCycler 480 SYBR Green I Master with UDG (Roche). In the PCR reaction (10 μl), 1 μl cDNA template was mixed with 200 nM primers and 5 μl SYBR PCR master mix (LifeTech). The PCR conditions included incubation at 50℃ for 2 min to activate UDG, 95℃ for 2 min to activate Taq polymerase, and 40 cycles of 95℃ for 15 sec and

60℃ for 1 min. Melting curves were generated after each PCR run to evaluate the size of PCR products. Each sample was tested in triplicate, and the mean Ct value was used for analysis if the coefficient of variation was <10%. If not, the mean of two closest reactions was used. As an internal reference, *GAPDH* expression was measured simultaneously with *TOPORS-AS1* in all the tumor samples. *CTNNB1* expression (mRNA for β-catenin) was also analyzed with RT-qPCR. Primer sequences for *TOPORS-AS1* amplification are shown in Supplemental Table S1 together with other PCR primers used in the study.

Plasmid amplification

For amplification, the *TOPORS-AS1* plasmid (50 ng) was gently mixed with 25 µl competent E. coli cells (#C404010, Thermo Fisher Scientific) for 30 minutes on ice, and then the mixture was heat shocked for 90 seconds in a water bath at 42℃. After incubation on ice for 2 minutes, 250 µl LB media was added into the mixture and shaken at 250 rpm for one hour. Appropriate antibiotics were added into the mixture, and 100 µl of the mixture was spread onto the LB plates which were further incubated overnight at 37℃. After that, the right colonies were selected and further incubated for plasmid extraction, using the PureLinkTM HiPure Plasmid RFilter Midiprep kit (#K210014, Thermo Fisher Scientific) according to the manufacturer's instructions.

Plasmid transfection and cell selection

The transfected cells were incubated for 48 hours, and then the culture was replaced with fresh medium that contained puromycin (0.5 µg/ml for SKOV3, 0.4 µg/ml for IGROV1). The puromycin-containing medium was changed every 3 days until cells stably expressing *TOPORS-AS1* were established. A single cell clone was also isolated from the stable cell pool and

developed through the limiting dilution cloning. *TOPORS-AS1* expression in the cells was measured with RT-qPCR after three weeks of transfection. The AllPrep DNA/RNA kit (Qiagen) was used for total RNA extraction, and the RNA was reverse-transcribed to cDNA using the cDNA Reverse Transcription kit (Themo Fisher Scientific).

ChIP assay

After 48 hours of plasmid transfection, formaldehyde at a final concentration 1% was added to the cell culture for crosslinking DNA and histone. After incubation at 37°C for 10 minutes, cells were washed twice with cold PBS containing protease inhibitors (1 mM phenylmethylsulfonyl fluoride, 1 µg/mL aprotinin, and 1 µg/mL pepstatin A). Cell lysates were sonicated to shear DNA into lengths between 200 and 1000 base pairs. Sonicated nuclear fraction was incubated overnight at 4°C on rotation with anti-VDR antibody (#12550 from Cell Signaling Technology) or anti-IgG antibody (#12-370 from EMD Millipore) as a control. The materials were further mixed with 60 µl of Protein A Agarose/Salmon Sperm DNA (50% Slurry) for one hour of incubation on rotation at 4°C. The antibody/histone complexes were collected in a buffer (1%SDS, 0.1M NaHCO3) which was further mixed with 20 µl of 5 M NaCl. The solution was heated to 65°C and incubated for 4 hours to break the histone-DNA crosslink. Ten µl of 0.5 M EDTA, 20 µl of 1 M Tris-HCl, pH 6.5, and 2 µl of 10 mg/ml Proteinase K were added and the mixtures were incubated for one hour at 45°C. DNA was recovered by phenol/chloroform extraction followed by ethanol precipitation. Enriched *TOPORS-AS1* promoters in the samples were measured by qPCR using the primers listed in Supplemental Table S1. Each assay was performed in triplicate and each experiment was repeated 3 times.

Vitamin D receptor upregulates lncRNA *TOPORS-AS1* which inhibits the Wnt/β-catenin pathway and associates with favorable prognosis of ovarian cancer

Short title: *TOPORS-AS1*, β-catenin, and VDR in ovarian cancer

Yuanyuan Fu[1, 4], Dionyssios Katsaros[2], Nicoletta Biglia[3], Zhanwei Wang[1], Ian Pagano[1], Marcus Tius[1], Maarit Tiirikainen[1], Charles Rosser[5], Haining Yang[1], Herbert Yu[1, *]

Figure 3D

IGROV1

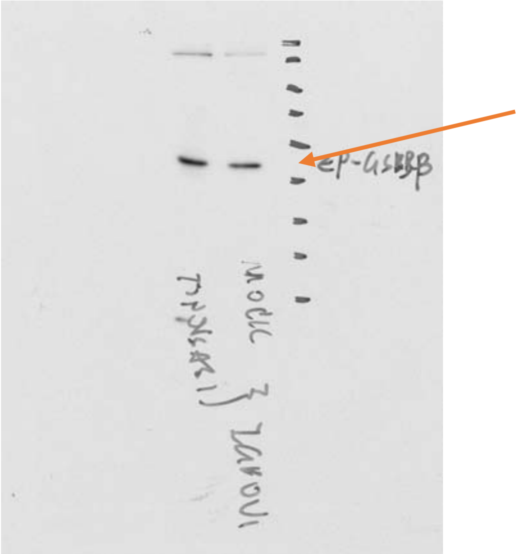

p-GSK3β (Y216)

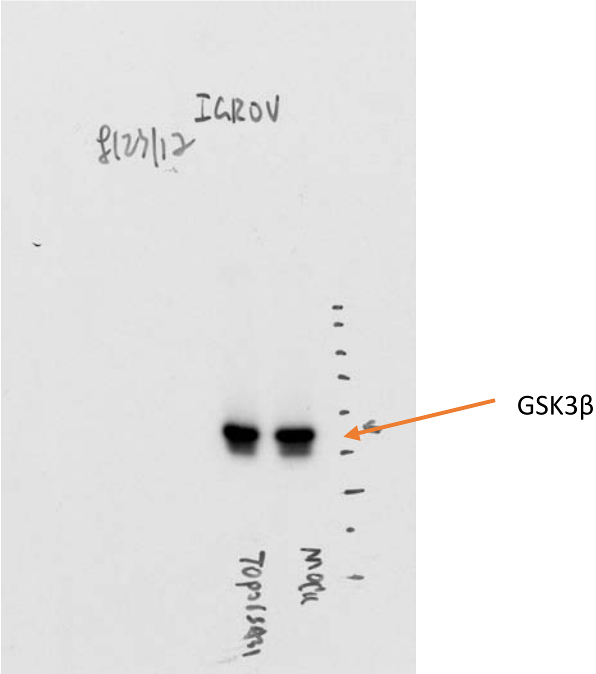

GSK3β

p-β-catenin(ser33/37/thr41)

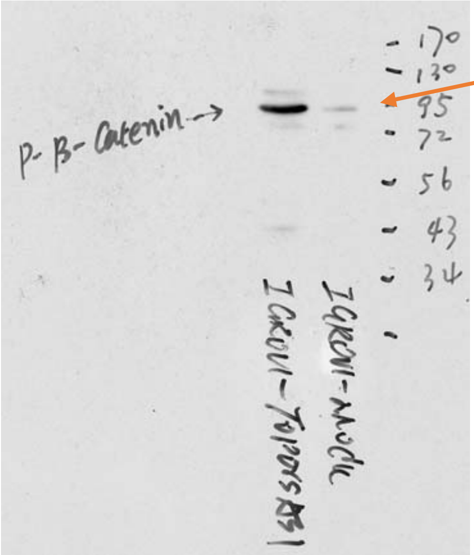

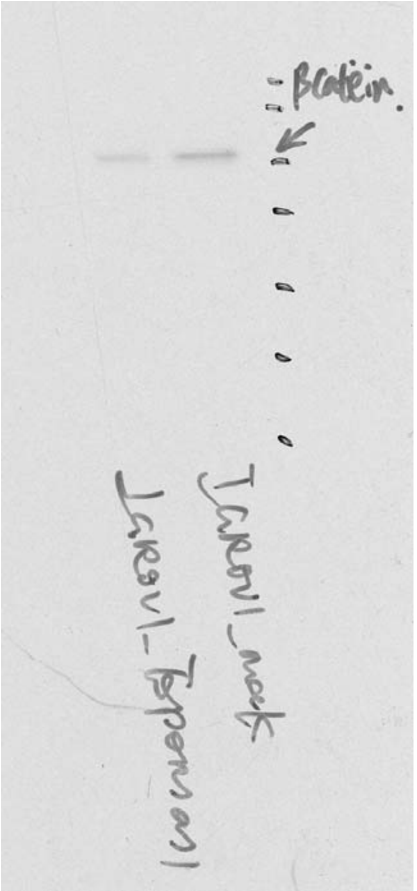

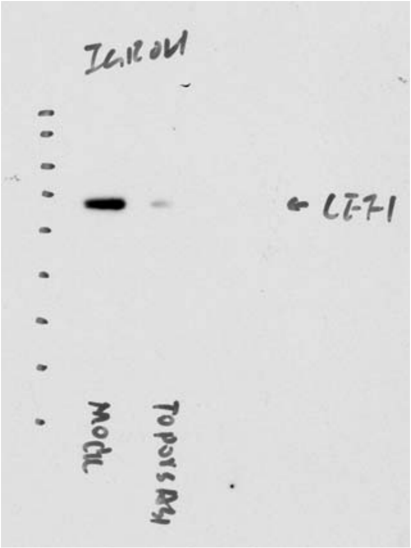

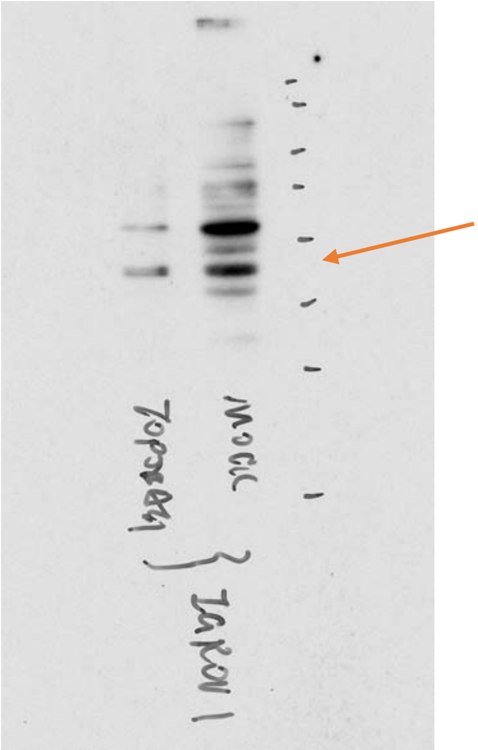

TCF1/TCF7

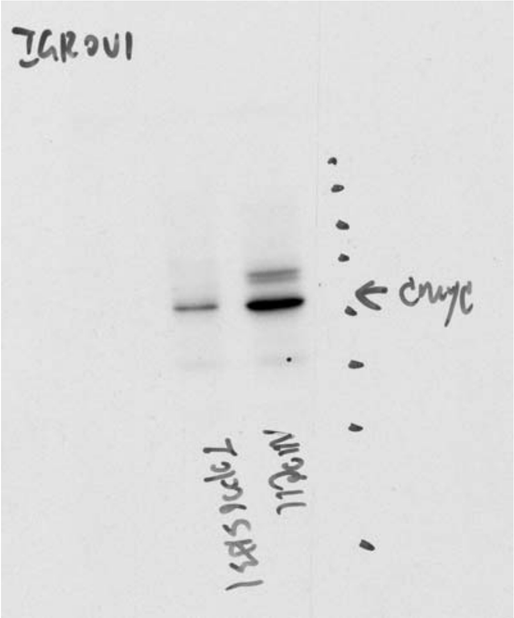

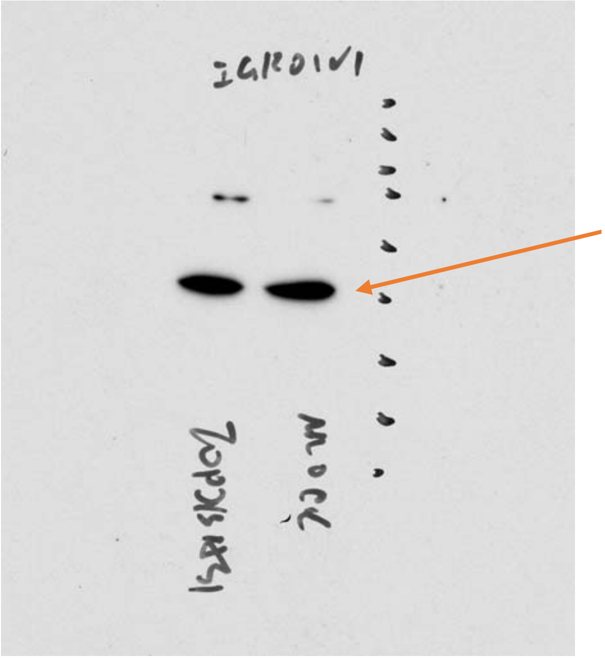

β-actin

SKOV3

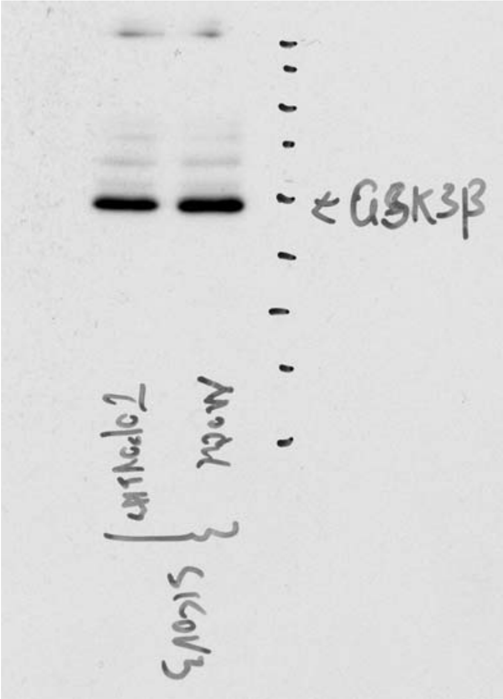

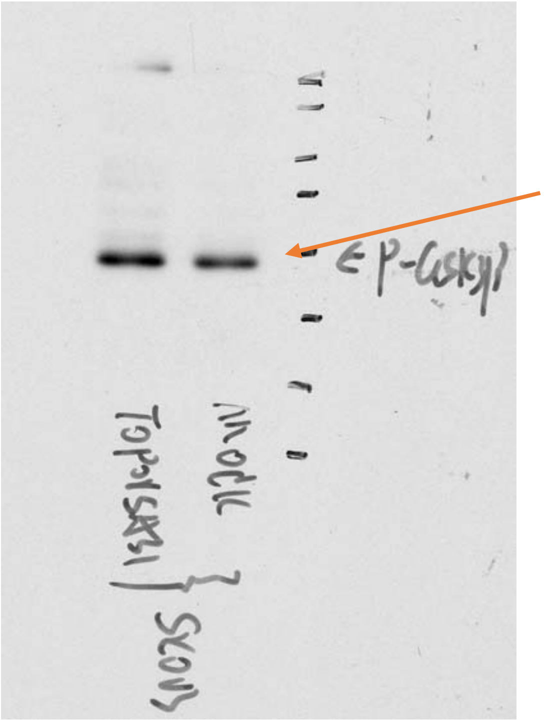

p-GSK3β (Y216)

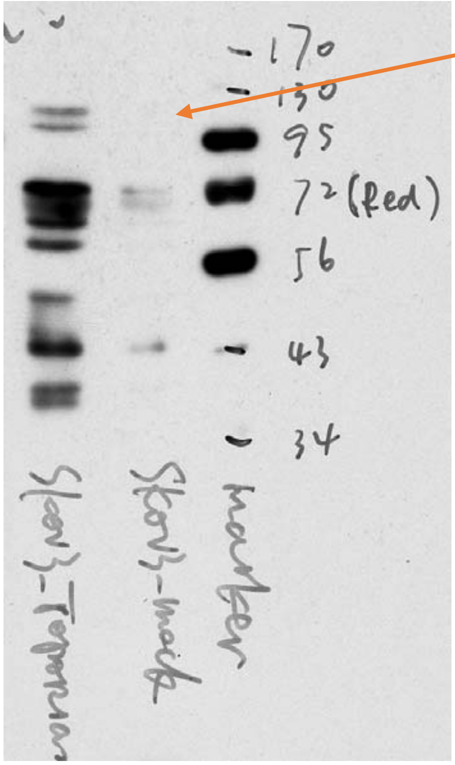

p-β-catenin(ser33/37/thr41)

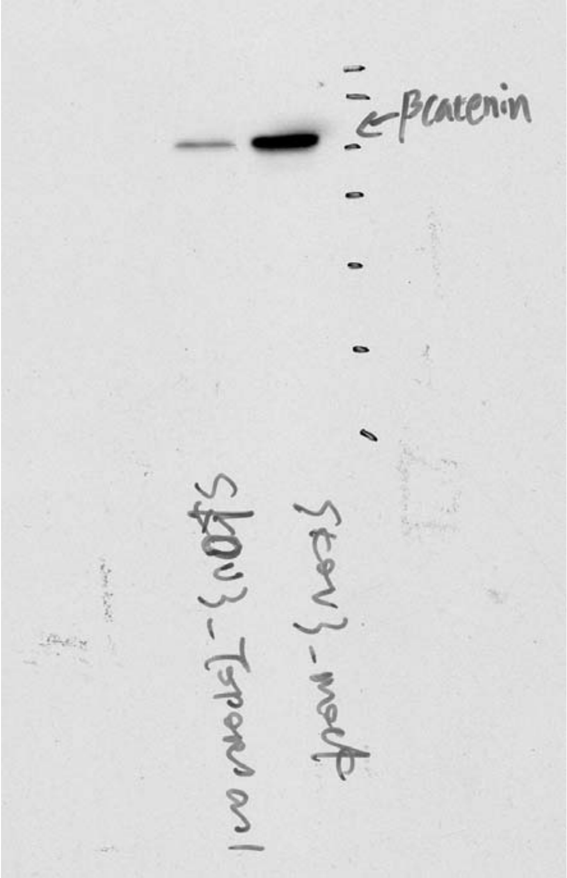

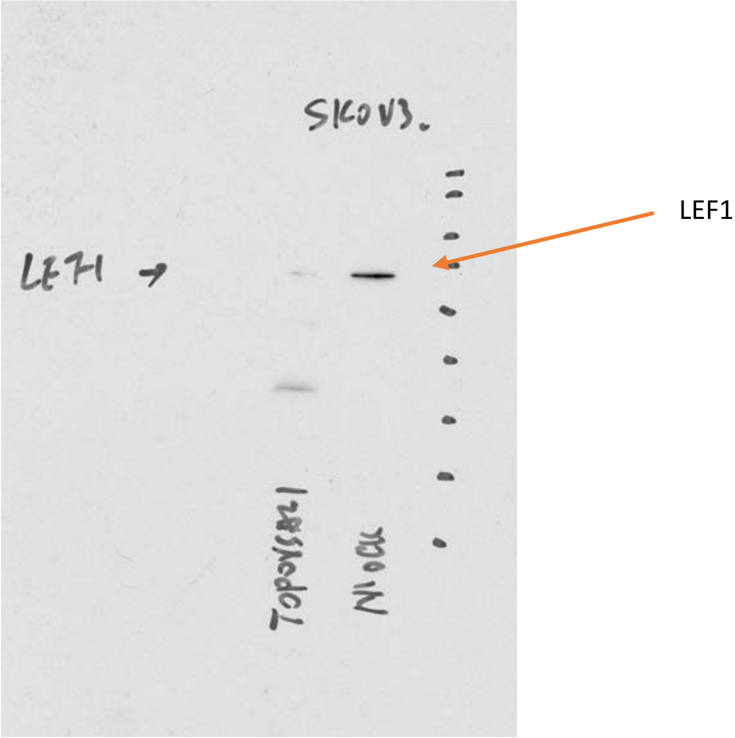

LEF1

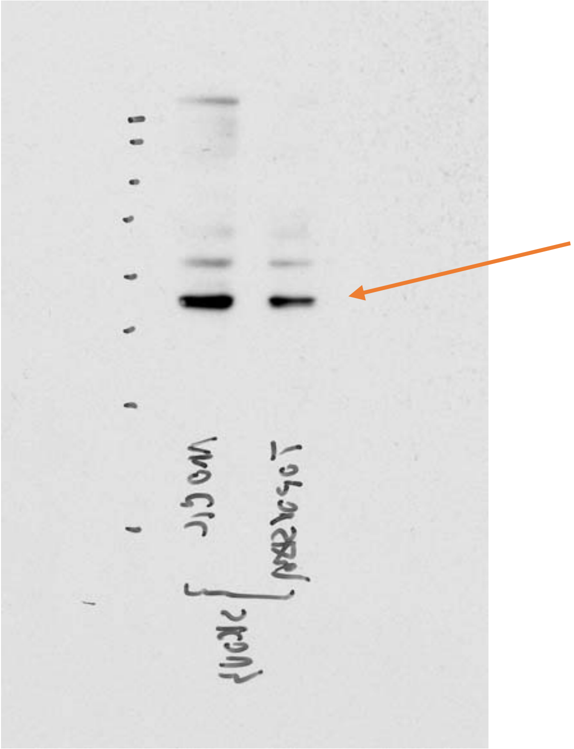

TCF1/TCF7

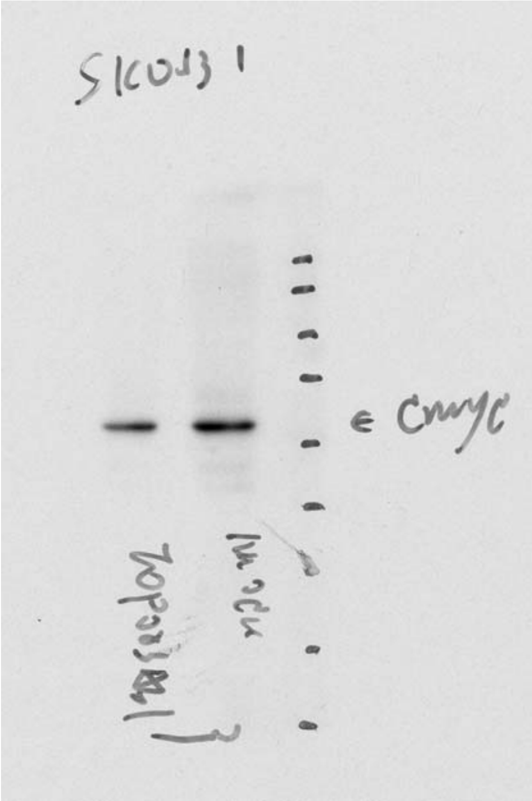

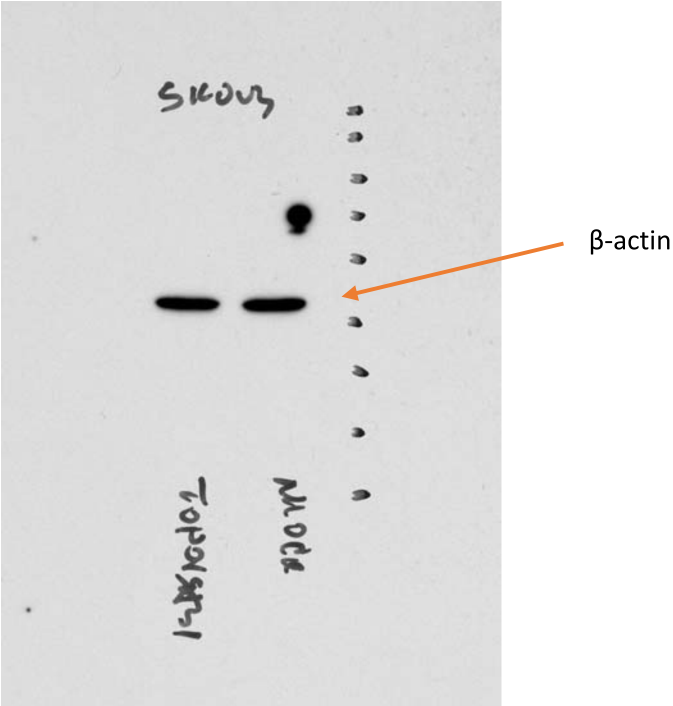

β-actin

Figure 3E

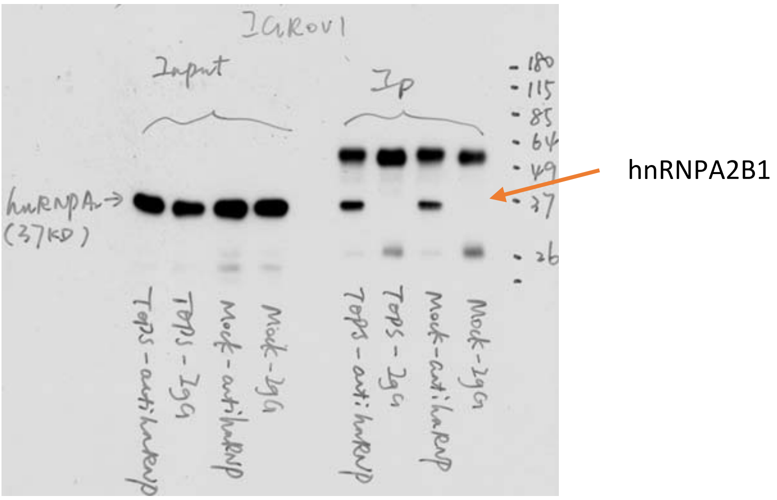

Figure 3G

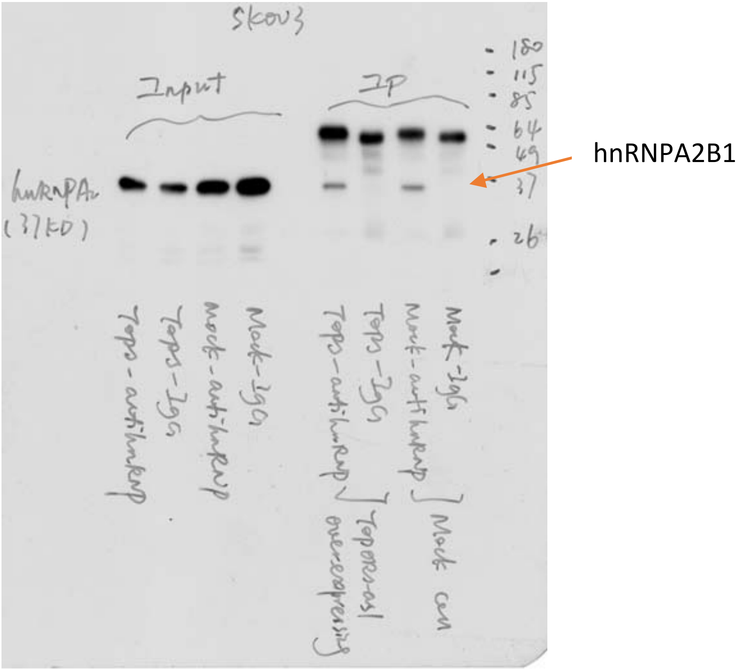

Figure 3I

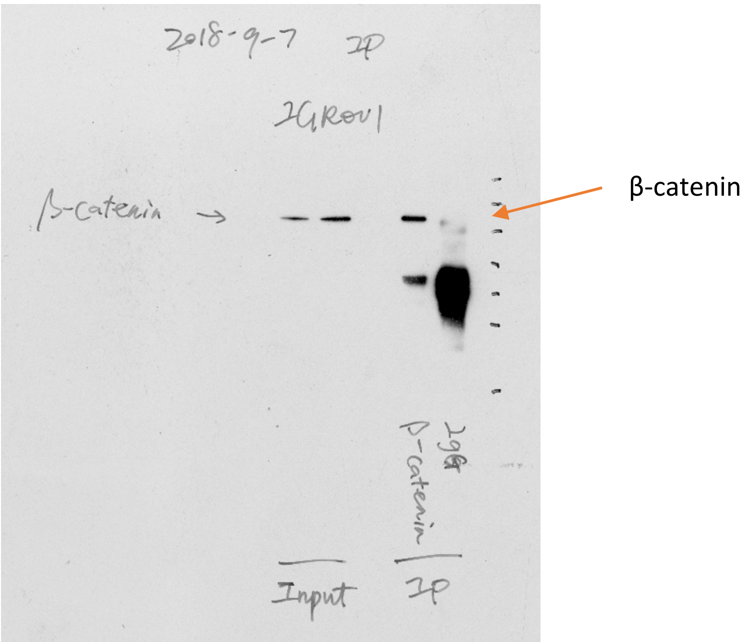
2018-9-7 IP
IGROV1
β-catenin
β-catenin
IgG
Input
IP
β-catenin

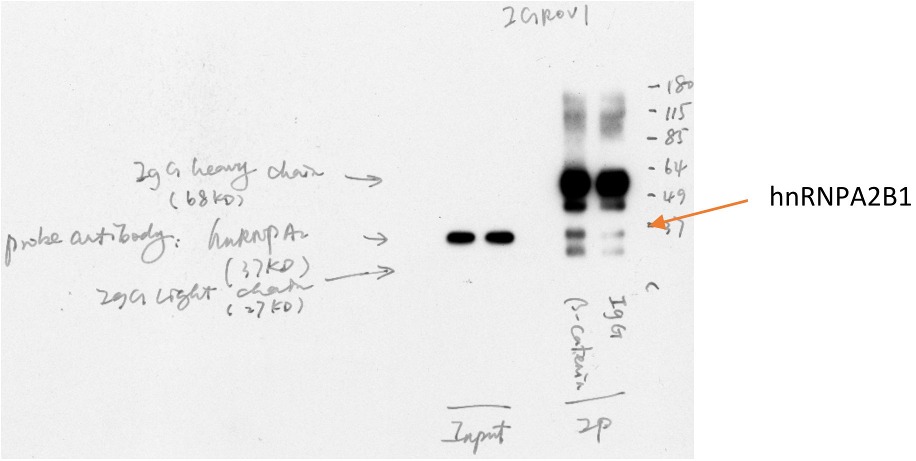
IGROV1
180
115
85
64
49
37
IgG heavy chain
(68KD)
probe antibody: hnRNPA2
(37KD)
IgG light chain
(27KD)
β-catenin
IgG
Input
IP
hnRNPA2B1

Figure 3J

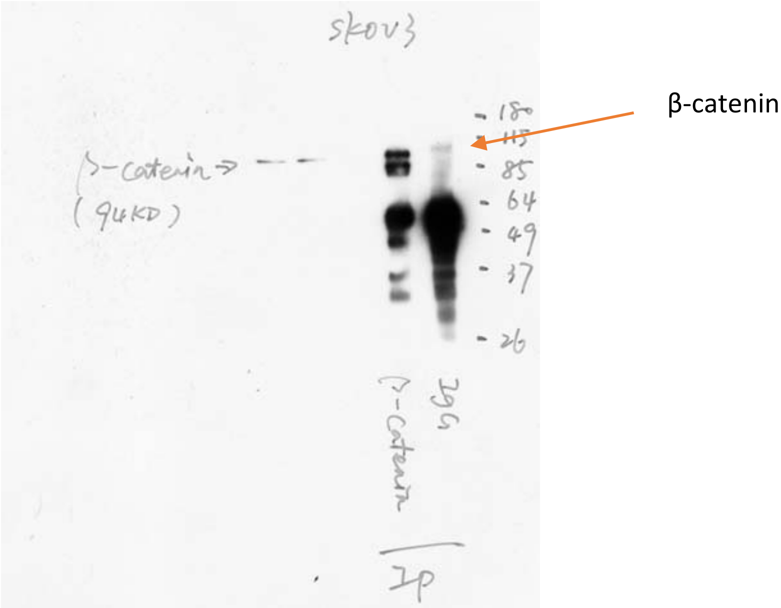

β-catenin

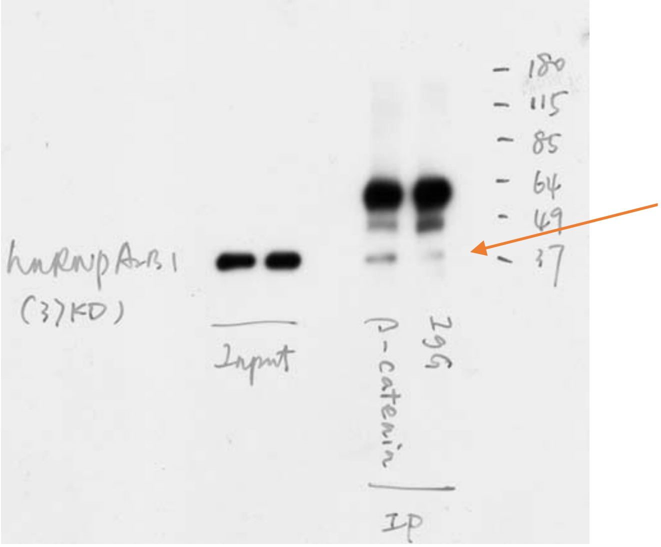

hnRNPA2B1

Figure 3K

IGROV1

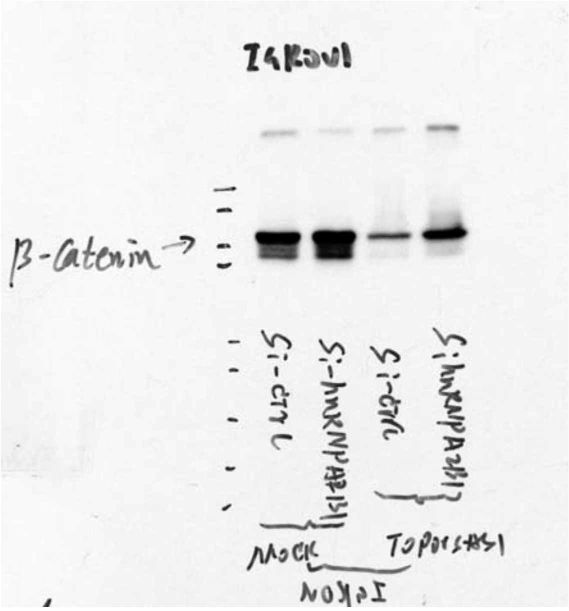

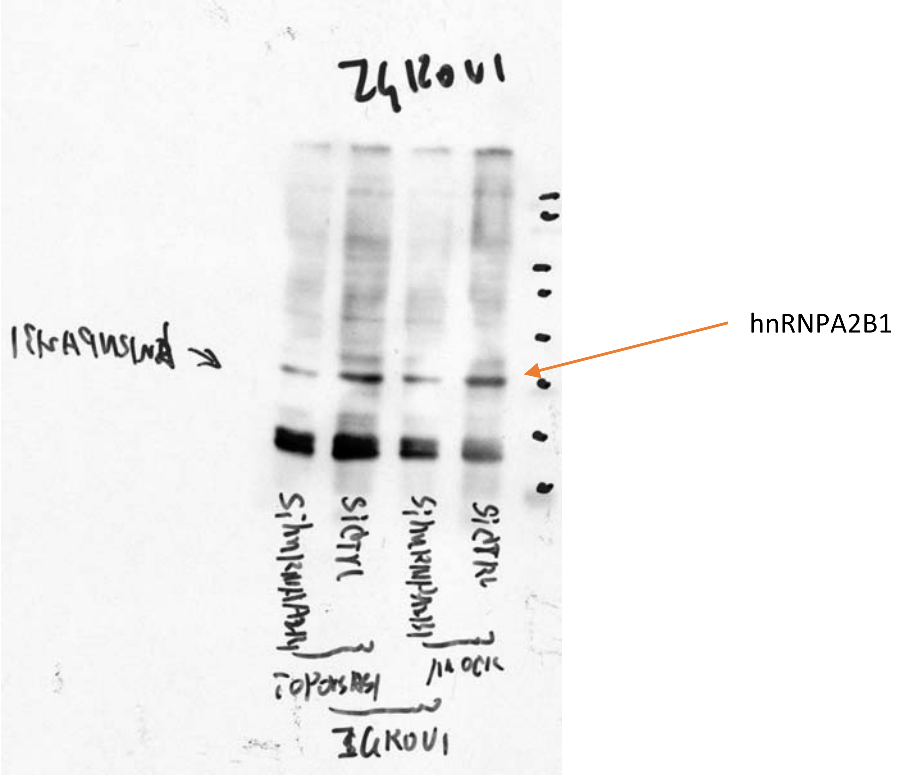

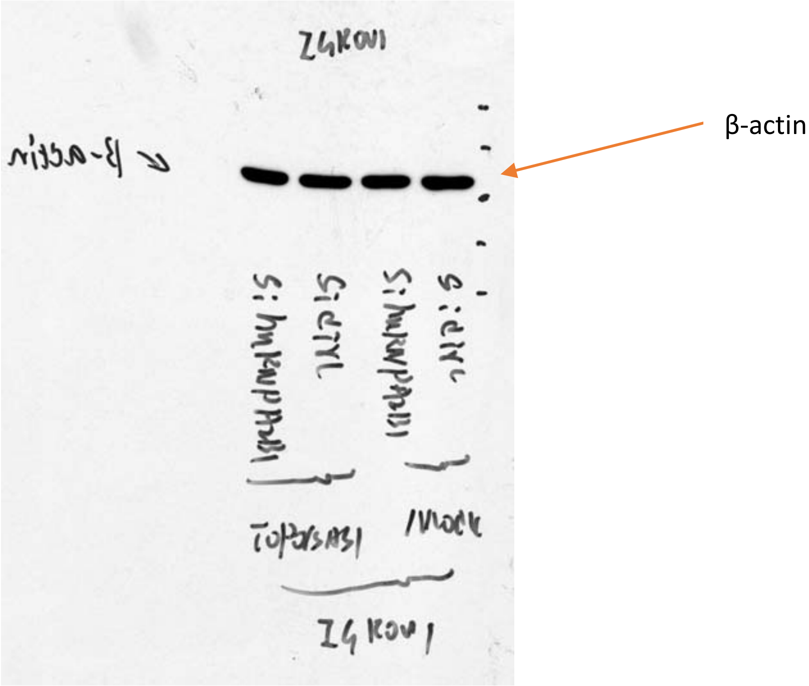

β-actin

SKOV3

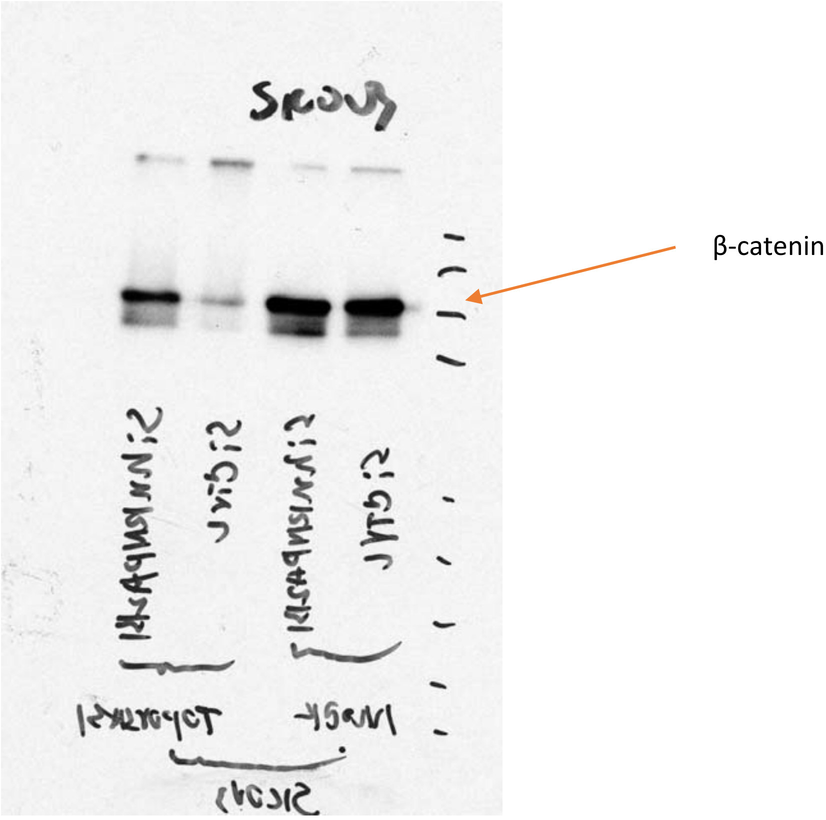

β-catenin

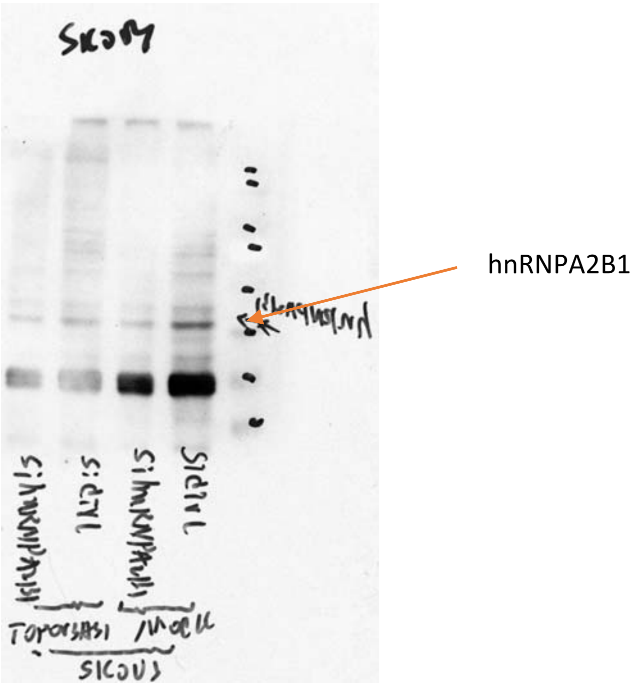
hnRNPA2B1

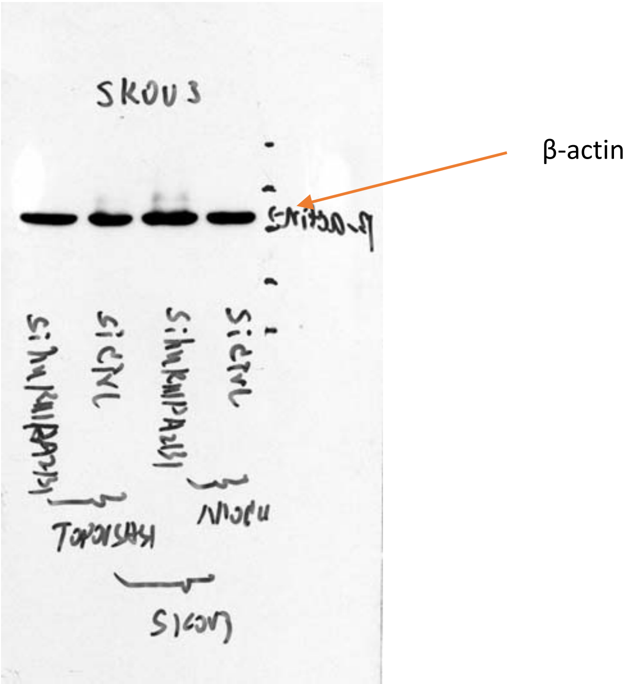
β-actin

Figure 4B

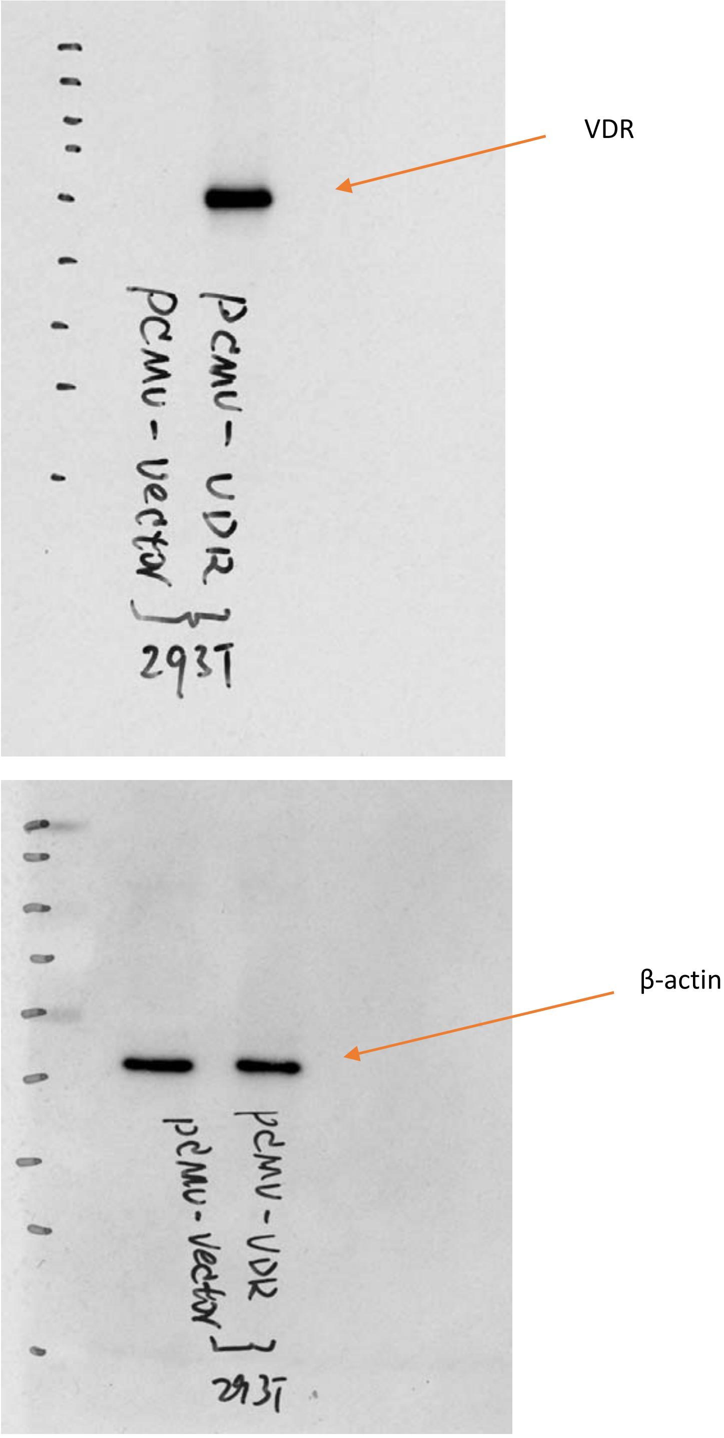
VDR
pCMV-VDR
pCMV-Vector
293T
β-actin
pCMV-VDR
pCMV-Vector
293T

Figure 4E

IGROV1

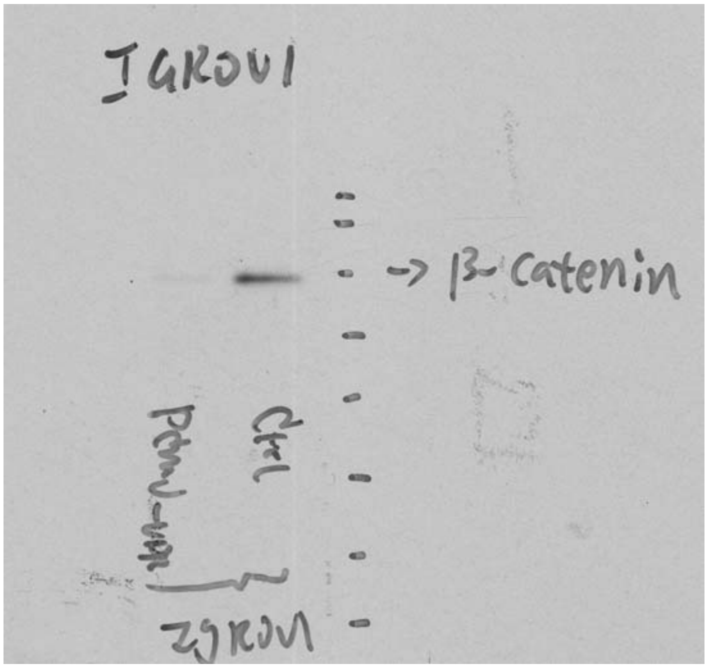

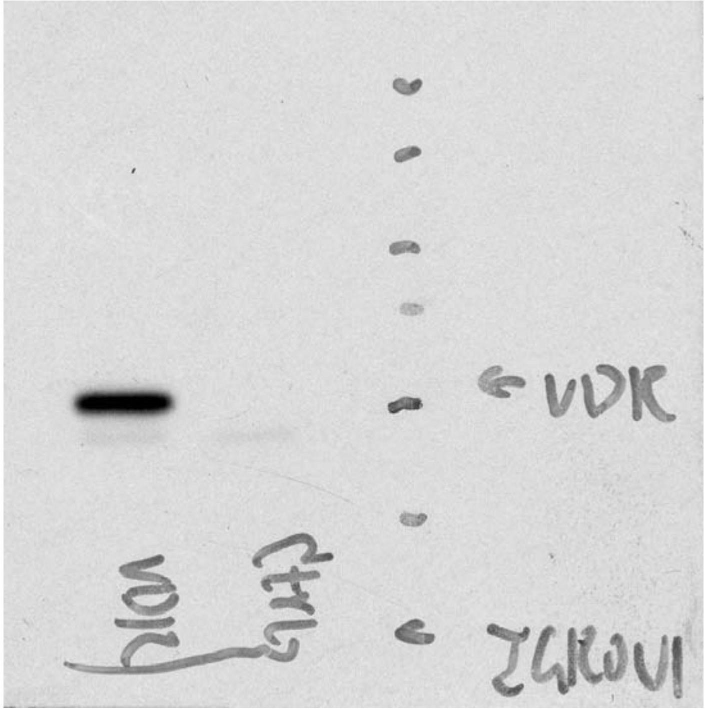

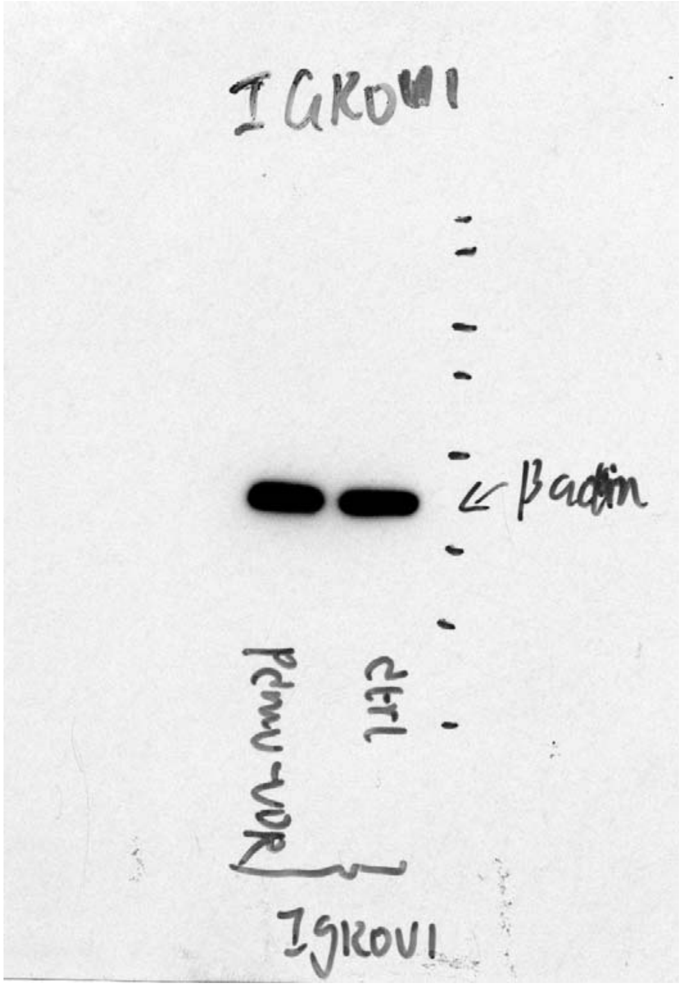
IGROV1
β actin
Pcmv-VDR
ctrl
IGROV1

SKOV3

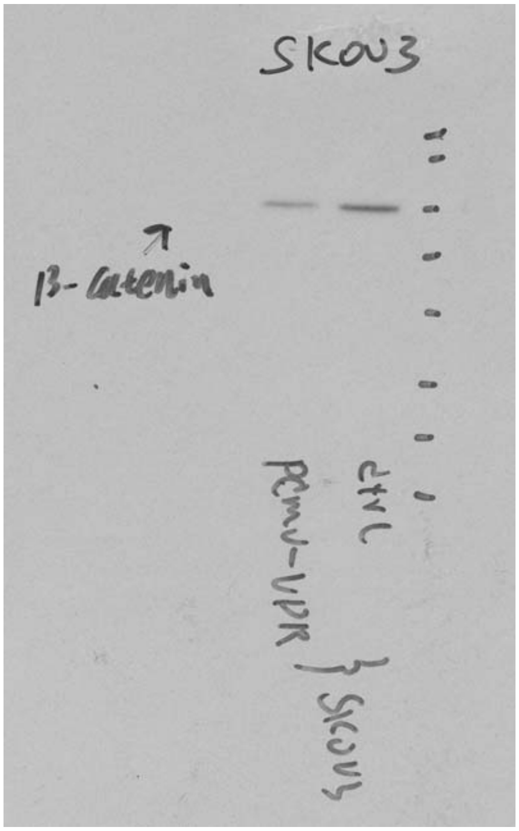

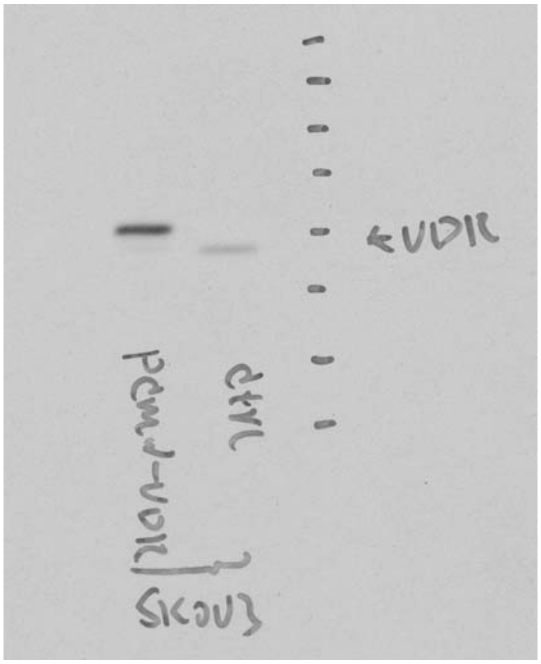

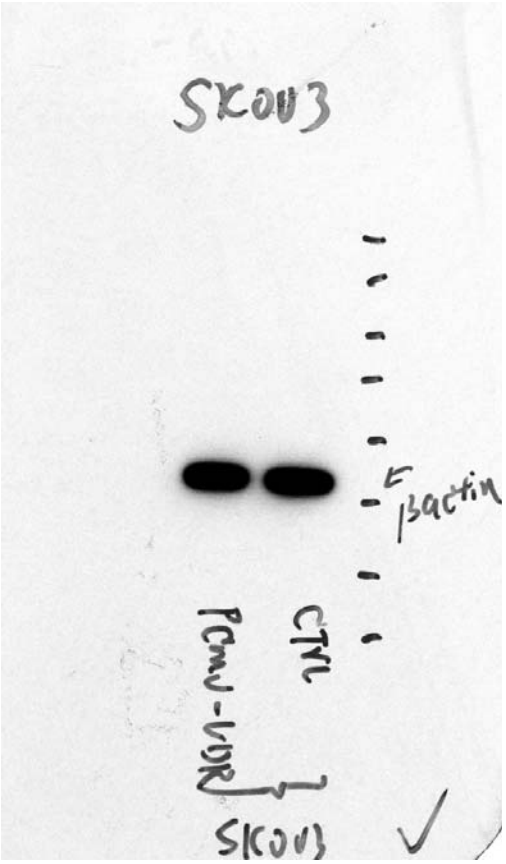
SKOV3
βactin
CTRL
PCMV-VDR
SKOV3

Figure 5A

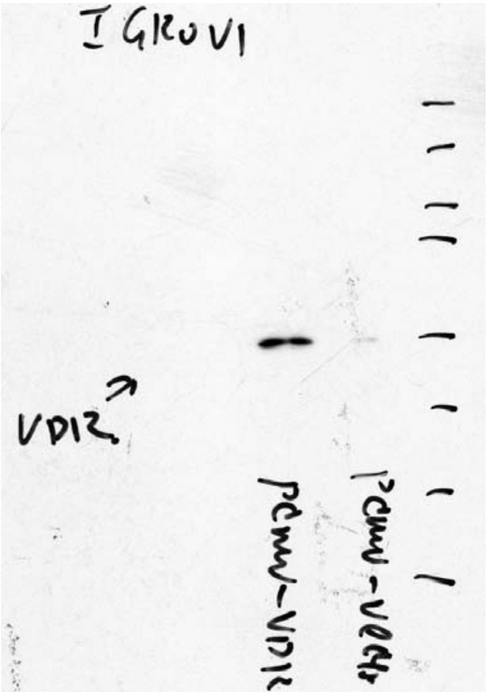
IGROV1
VDR
pCMV-VDR
pCMV-vector

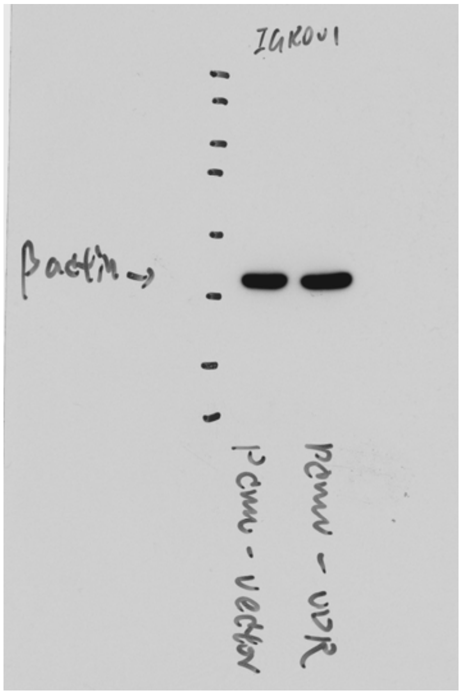
IGROV1
βactin
pCMV - vector
pCMV - VDR

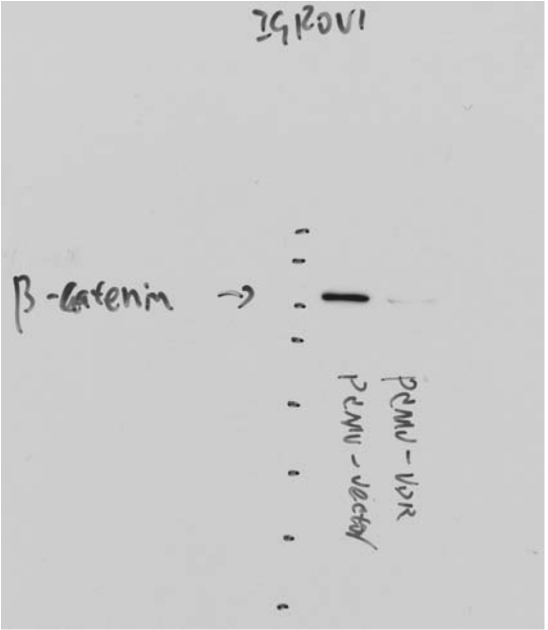
IGROV1
β-catenin →
pCMV-vector
pCMV-VDR

Figure 5B

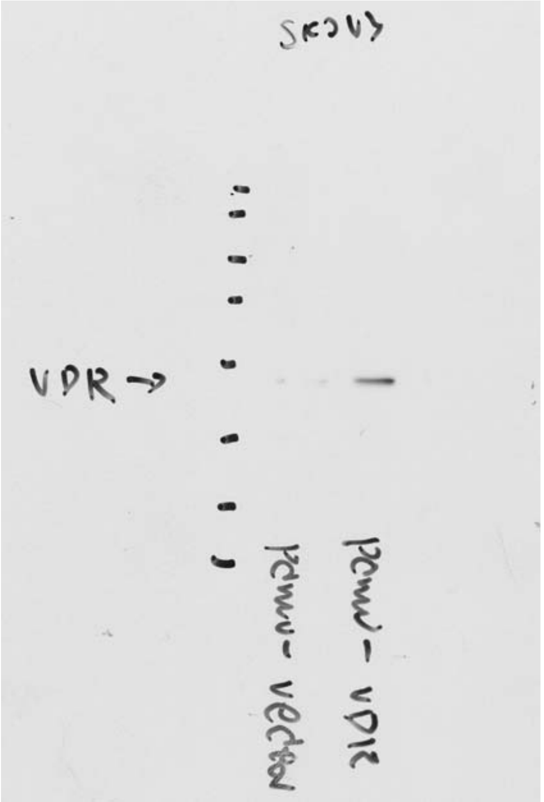
SKOV3
VDR →
pCMV-vector
pCMV-VDR

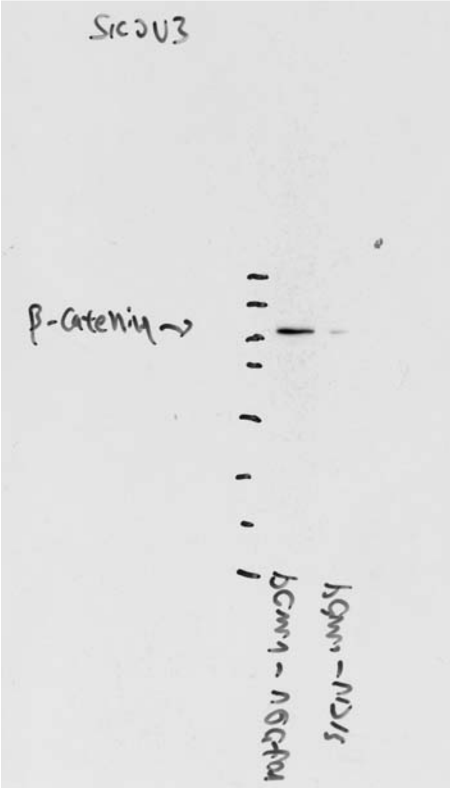

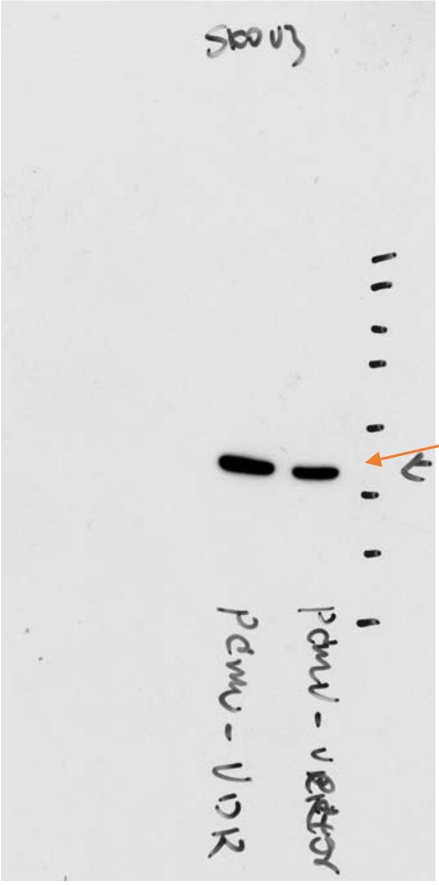

β-actin

Figure 5E

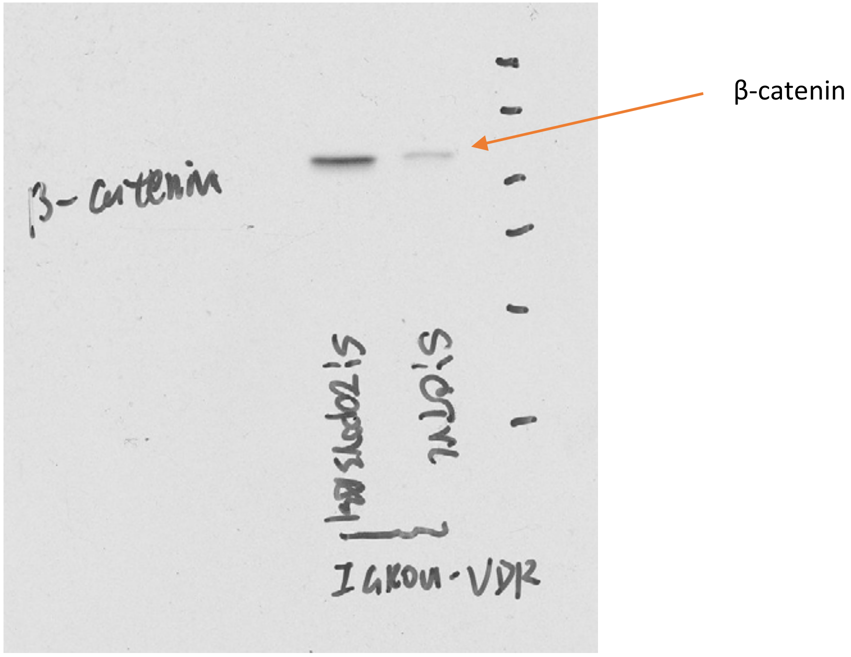

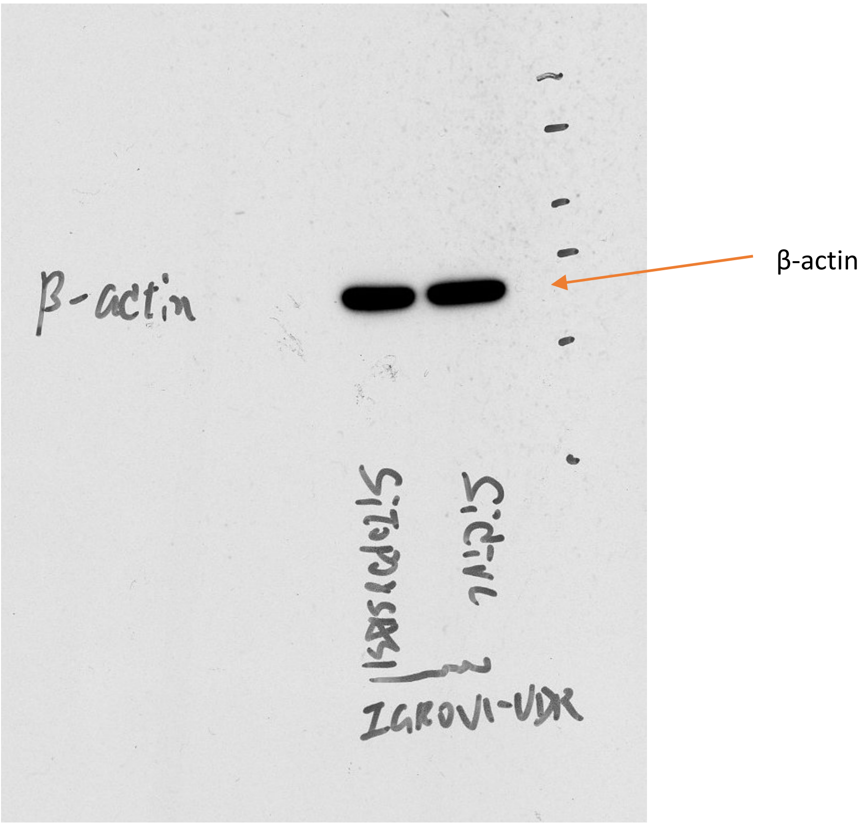

Figure 5F

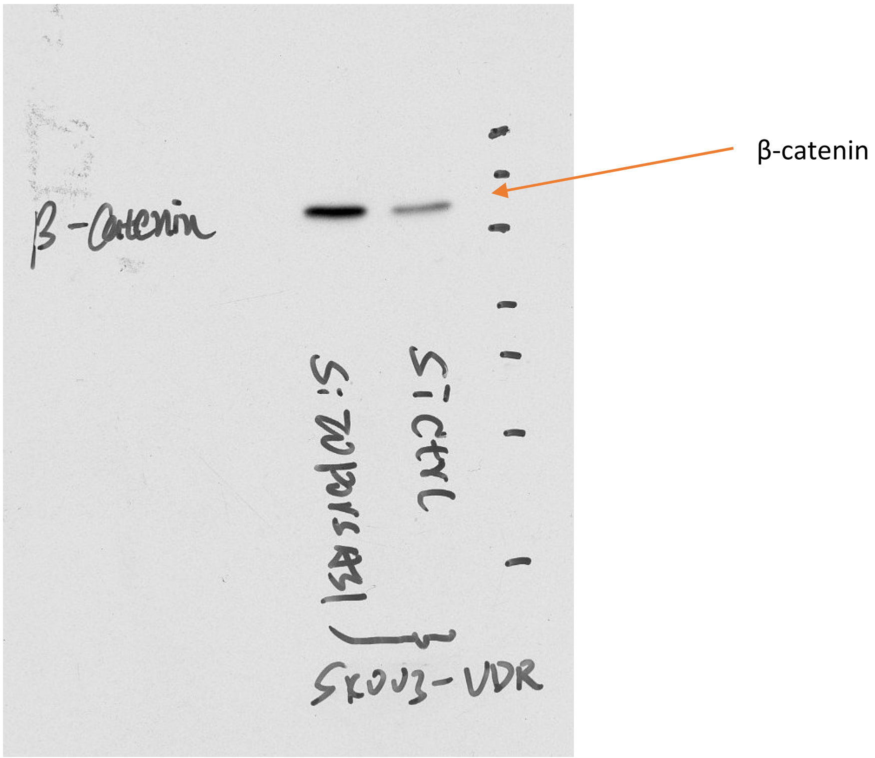

β-catenin
β-Catenin
SiCtrl
SKOV3-VDR

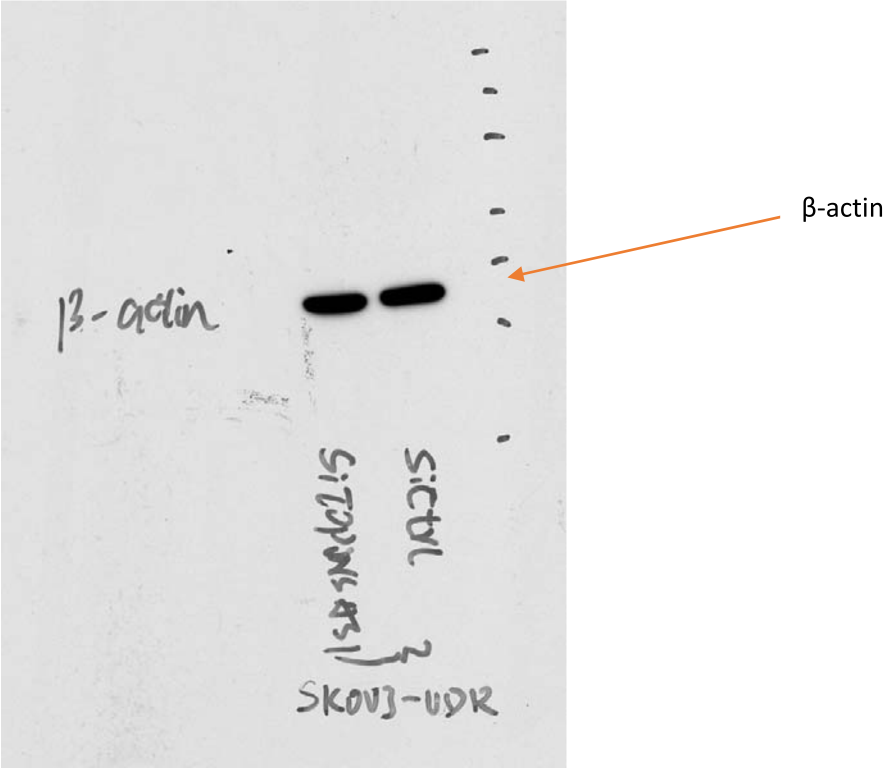

β-actin
β-actin
SiCtrl
SKOV3-VDR

Figure 5J

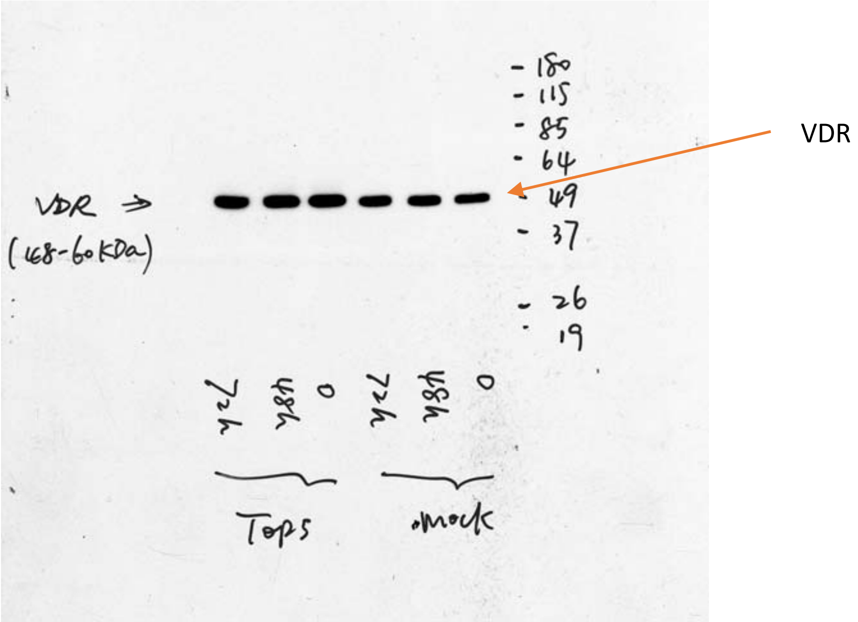
VDR →
(48-60KDa)
180
115
85
64
49
37
26
19
72h
48h
0
72h
48h
0
Top3
Mock
VDR

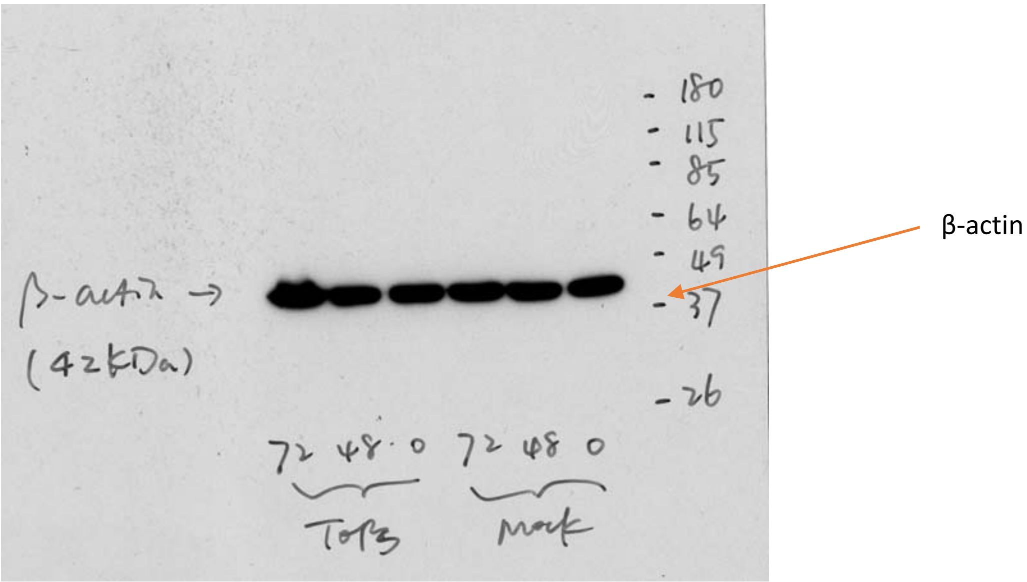
β-actin →
(42KDa)
180
115
85
64
49
37
26
72
48
0
72
48
0
Top3
Mock
β-actin

5L

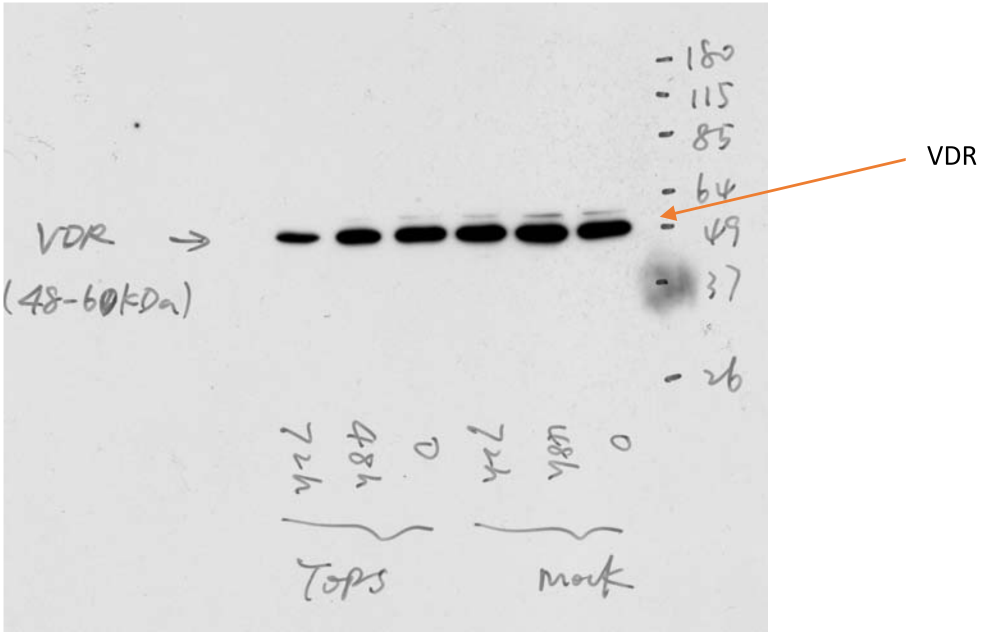

VDR →
(48-60KDa)
180
115
85
64
49
37
26
72h
48h
0
72h
48h
0
Tops
Mock
VDR

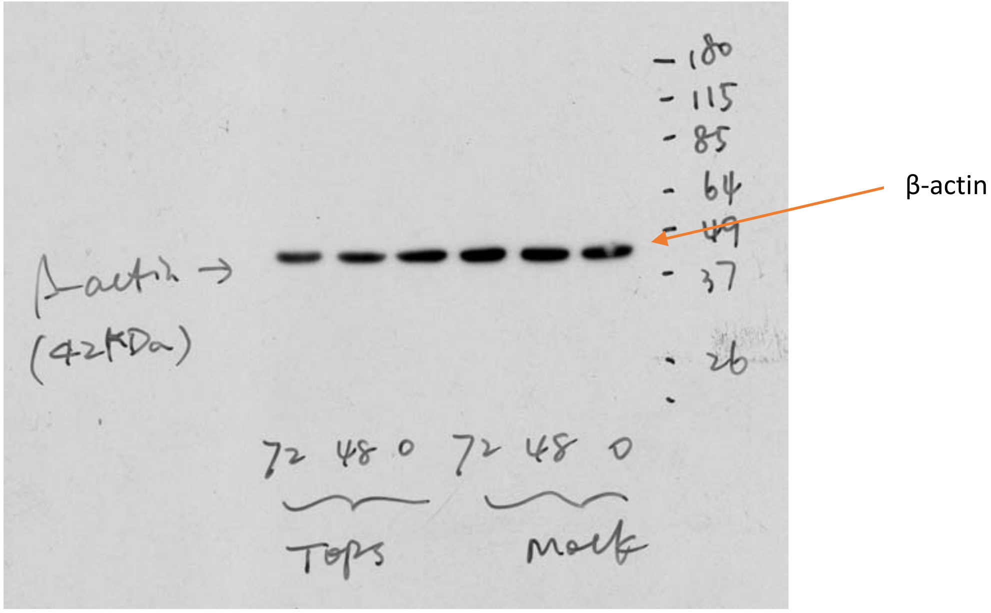

β-actin →
(42KDa)
180
115
85
64
49
37
26
72
48
0
72
48
0
Tops
Mock
β-actin
